# SETD1B-mediated broad H3K4me3 controls proper temporal patterns of gene expression critical for spermatid development

**DOI:** 10.1038/s41422-025-01080-0

**Published:** 2025-03-04

**Authors:** Zhen Lin, Bowen Rong, Ruitu Lyu, Yuxuan Zheng, Yao Chen, Junyi Yan, Meixia Wu, Xiaogang Gao, Fuchou Tang, Fei Lan, Ming-Han Tong

**Affiliations:** 1 Key Laboratory of Multi-Cell System, Shanghai Key Laboratory of Molecular Andrology, Shanghai Institute of Biochemistry and Cell Biology, Center for Excellence in Molecular Cell Science, Chinese Academy of Sciences, University of Chinese Academy of Sciences, Shanghai, China; 2 Shanghai Key Laboratory of Medical Epigenetics, State International Co-laboratory of Medical Epigenetics and Metabolism, Institutes of Biomedical Sciences, Fudan University, and Key Laboratory of Carcinogenesis and Cancer Invasion, Ministry of Education, Liver Cancer Institute, Zhongshan Hospital, Fudan University, Shanghai, China; 3 Biomedical Pioneering Innovation Center, School of Life Sciences, Peking-Tsinghua Center for Life Sciences, Peking University, Beijing, China; 4 Department of Organ Transplantation, Changhai Hospital, Naval Military Medical University, Shanghai, China

**Keywords:** Developmental biology, Epigenetics

## Abstract

Epigenetic programming governs cell fate determination during development through intricately controlling sequential gene activation and repression. Although H3K4me3 is widely recognized as a hallmark of gene activation, its role in modulating transcription output and timing within a continuously developing system remains poorly understood. In this study, we provide a detailed characterization of the epigenomic landscapes in developing male germ cells. We identified thousands of spermatid-specific broad H3K4me3 domains regulated by the SETD1B-RFX2 axis, representing a previously underappreciated form of H3K4me3. These domains, overlapping with H3K27ac-marked enhancers and promoters, play critical roles in orchestrating robust transcription and accurate temporal control of gene expression. Mechanistically, these broad H3K4me3 compete effectively with regular H3K4me3 for transcriptional machinery, thereby ensuring robust levels and precise timing of master gene expression in mouse spermiogenesis. Disruption of this mechanism compromises the accuracy of transcription dosage and timing, ultimately impairing spermiogenesis. Additionally, we unveil remarkable changes in the distribution of heterochromatin marks, including H3K27me3 and H3K9me2, during the mitosis-to-meiosis transition and completion of meiotic recombination, which closely correlates with gene silencing. This work underscores the highly orchestrated epigenetic regulation in spermatogenesis, highlighting the previously unrecognized role of *Setd1b* in the formation of broad H3K4me3 domains and transcriptional control, and provides an invaluable resource for future studies toward the elucidation of spermatogenesis.

## Introduction

The germline possesses a unique ability to generate a new individual and is paramount for transmitting a complete genome and proper epigenetic information to subsequent generations. Male germ cell development, known as spermatogenesis, comprises a complex series of differentiation stages. These include mitotic expansions of spermatogonia, meiotic divisions of spermatocytes, and morphological transformations of spermatids to generate haploid sperm that contribute to the totipotency of a zygote at fertilization.^1,2^ During spermatogenesis, male germ cells express the most complex and diverse transcriptome of all mammalian tissues.^3,4^ The developmental regulation of gene expression programs is tightly governed by a complex interplay between genetic and epigenetic factors, with chromatin epigenetic states closely associated with transcriptional regulation. For instance, the transcription factor MYBL1 acts as a master regulator of male meiosis, while transcription factors RFX2, CREM, and SOX30 control spermatid development.^4–10^ Genetic disruption of epigenetic regulators has shown that impaired DNA methylation, histone modification, or nucleosome remodeling leads to male infertility, developmental defects, and health problems in offspring.^11–19^ These results underline the importance of understanding epigenetic regulation and the interplay between transcription factors and epigenetic factors during spermatogenesis.

The well-defined cell division, differentiation, and development associated with mammalian spermatogenesis make it an ideal system for investigating the pivotal functions and molecular mechanisms linked to epigenetic programming. Over the last decade, significant efforts have been dedicated to establishing genome-wide epigenetic profiles in several stages of heterogeneous male germ cells, providing important insights into epigenetic regulation underlying spermatogenesis.^20–26^ Despite this progress, achieving the epigenome of male germ cells at defined substages remains challenging due to difficulties in purifying homogeneous populations of specific substage of male germ cells from testes.^20–26^ Notably, it is still unclear how, or even whether, specific chromatin states help establish stage-specific transcriptomes in male germ cells, particularly concerning genome-wide gene repression during the mitotic-to-meiotic transition and gene reactivation during the meiotic-to-post-meiotic transition.

Histone H3 trimethylated at lysine 4 (H3K4me3) is typically enriched at active or bivalent promoters and generally exists in a sharp form spanning 1–2 kb.^27–30^ While earlier studies argued against a link between H3K4me3 and transcription,^31,32^ recent studies suggest that H3K4me3 plays key roles in Pol II function and transcription.^33,34^ Indeed, this modification has been reported to be involved in the formation of the transcriptional preinitiation complex (PIC) through its interaction with TAF3, a component of the general transcriptional factor IID (TFIID) complex.^35,36^ Recently, broad H3K4me3 domains have been identified in different cell types, including certain somatic cell types and preimplantation embryonic cells.^37–41^ These domains are believed to be associated with high gene expression that maintains cell identity,^37,38,40^ while it has also been reported that the large H3K4me3 domains (over 10 kb) found in oocytes are transcriptionally silent.^39,41^ How broad H3K4me3 domains are established and how they affect transcriptional outputs to influence diverse cellular function during development still remain largely unclear. SETD1B/KMT2G, an understudied H3K4me3 methyltransferase, has recently been shown to regulate H3K4me3 s in mouse embryonic stem cells and neuronal development.^42,43^ The distinct mechanisms by which SETD1B acts compared to other H3K4 methyltransferases, i.e., KMT2A-D and KMT2F, remain elusive. Furthermore, H3K4me3, especially broad H3K4me3, was found to colocalize with H3K27ac-marked enhancers during oogenesis.^44,45^ However, the H3K4me3 dynamics during spermiogenesis remains unclear.

Here, we systematically map chromatin states at 11 different spermatogenic stages to generate a comprehensive atlas of the changing epigenomic landscape in developing male germ cells. Our map covers major mitotic, meiotic, and post-meiotic stages during mouse spermatogenesis. The resulting information on histone modifications, DNA methylation, nucleosome occupancy, and transcriptomics provides deep insights into epigenomic and genomic mechanisms that control gene expression and cell differentiation. We uncover previously unknown broad H3K4me3 domains that each span over 5 kb and are consistently present during spermiogenesis. These domains that bookmark spermatid-specific genes are more widespread than super-enhancers (SEs). Loss of *Setd1b* results in nearly complete depletion of broad H3K4me3. Subsequent analyses reveal that *Setd1b* depletion causes the redistribution of Pol II from broad H3K4me3 domains to regular H3K4me3 regions and compromises the output and timing of stage-specific gene expression, thereby impairing spermiogenesis. In addition, we find that repressive histone marks, namely H3K27me3, H3K9me2, and H3K9me3, distribute mutually exclusively during the mitosis-to-meiosis transition and completion of meiotic recombination and synapsis, correlating with global transcription output and retrotransposon and mitotic/meiotic gene silencing.

## Results

### Systematic profiling of chromatin epigenomic landscapes during mouse spermatogenesis

**Fig. 1:**
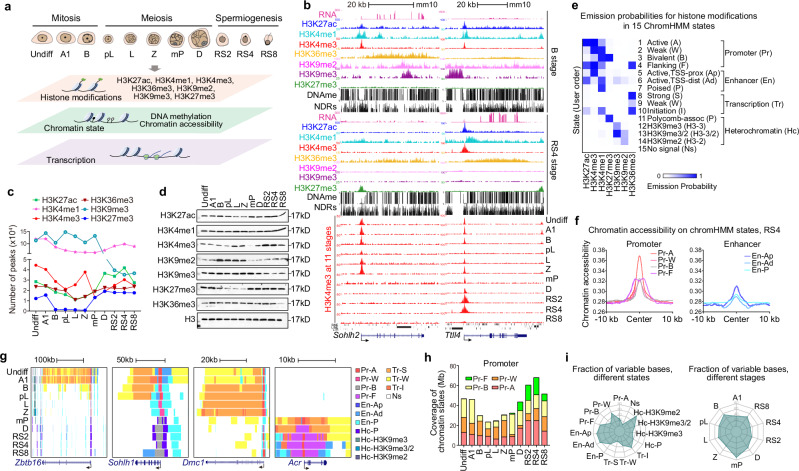
Establishment of a comprehensive chromatin epigenomic atlas during mouse spermatogenesis. **a** Schematic workflow showing the 11 subtypes of synchronized mouse spermatogenic cells isolated. These include mitotic stages (Undiff: undifferentiated spermatogonia, A1: type A1 spermatogonia, B: type B spermatogonia), meiotic stages (pL: preleptotene spermatocytes, L: leptotene spermatocytes, Z: zygotene spermatocytes, D: diplotene spermatocytes) and spermiogenesis stages (RS2: steps 1–2 round spermatids, RS4: steps 3–4 round spermatids, RS8: steps 7–8 round spermatids). Genome-wide landscapes of seven histone marks, nucleosome positioning and DNAme were profiled in the 11 subtypes by ChIP-seq and NOMe-seq, respectively. The transcriptome was established by RNA-seq. **b** Snapshots of UCSC genome browser showing RNA-seq, histone marks, DNA methylation, and chromatin accessibility at B (upper panels) and RS4 (middle panels) stages, and H3K4me3 at 11 different stages (lower panels) during mouse spermatogenesis at the *Sohlh2* and *Ttll4* gene loci. **c** Quantification representation of ChIP-seq peak numbers for seven histone modifications in 11 stages during mouse spermatogenesis. **d** Western blot analyses showing the global levels of histone marks during mouse spermatogenesis. H3 was used as the internal control. **e** Emission probabilities for histone modifications in 15 ChromHMM chromatin states, with a descriptive title of each chromatin state shown on the right. Active promoters are proximal to TSS, marked by hyper H3K4me3 and H3K27ac. Active enhancers, enriched in H3K27ac and H3K4me1, are distal from the TSS. Transcriptional elongation signatures were decorated with H3K36me3. Heterochromatin regions are typically associated with H3K9me2, H3K9me3, or H3K27me3 but lack active marks. The no signal (Ns) state is characterized by the absence of any histone modification. **f** Average chromatin accessibility on different chromatin states (Promoter and Enhancer) at RS4 stage. **g** Chromatin state profiles at the gene loci of *Zbtb16*, *Sohlh1*, *Dmc1* and *Acr* across 11 different stages. Pr, promoter; En, enhancer; Tr, transcription; Hc, heterochromatin. **h** Stacked chart showing the genomic coverage of 4 promoter-related chromatin states. **i** Radar charts showing the fraction of variable bases across different stages (left panel) and distinct chromatin states (right panel). Points on the lines in both radar charts represent the median values of the fraction of variable bases.

To better understand epigenetic dynamics during spermatogenesis, we isolated homogeneous populations of male germ cells representing 11 consecutive developmental stages that cover mitotic, meiotic, and post-meiotic cells using a previously established protocol^4^ and systematically analyzed epigenomic modifications (Fig. 1a). Cytological and immunostaining studies revealed a high degree of purity in all isolated substages of spermatogenic cells (Supplementary information, Fig. S1). We profiled H3K27ac, H3K4me1, H3K4me3, H3K36me3, H3K9me2, H3K9me3, and H3K27me3 in these populations using chromatin immunoprecipitation sequencing (ChIP-seq) (Supplementary information, Table S1). These 7 modifications allow the assessment of functional elements (i.e., promoters, enhancers and gene bodies) and active/inactive states (i.e., active, poised and repressed states) (Fig. 1a; Supplementary information, Fig. S2a). Pearson’s correlation coefficients demonstrated a high degree of reproducibility between biological replicates, and at least two replicates were performed for each experiment (Supplementary information, Fig. S2b). All ChIP-seq data sets were uniformly processed and subjected to rigorous quality control (Supplementary information, Fig. S2c and Table S2). We also profiled genome-wide DNA methylation (DNAme) and chromatin accessibility (Supplementary information, Fig. S2d, e), as well as transcriptome (Supplementary information, Fig. S2f) using Nucleosome Occupancy and Methylome sequencing (NOMe-seq) and RNA sequencing (RNA-seq) in each population (Fig. 1a; Supplementary information, Tables S1, S2).

Histone marks, including H3K4me3, H3K9me2, H3K27me3, H3K27ac changed dramatically across cell stages (Fig. 1b–d; Supplementary information, Fig. S2i, j). Similarly, the overall number of nucleosome-depleted regions (NDRs) also shifted dynamically at different stages of spermatogenesis (Supplementary information, Fig. S2g). Of all analyzed stages, the population entering meiosis (preleptotene spermatocytes, pL) contained the lowest number of NDRs and DNAme levels (Supplementary information, Fig. S2g, h). This is consistent with our and others’ previous reports regarding lower DNAme levels at the onset of meiosis.^46–48^ Further analyses, including Pearson’s correlation coefficients analysis (Supplementary information, Fig. S3a), Euclidean distance analysis (Supplementary information, Fig. S3b), principal component analysis (PCA) (Supplementary information, Fig. S3c), and read density profiling (Supplementary information, Fig. S3d), collectively offer diverse perspectives on understanding the dynamic changes in chromatin states during spermatogenesis.

These developmental chromatin profiles were consistent with known stage-specific gene functions. For example, the spatiotemporal expression of *Sohlh2*, a spermatogonial marker, was consistent with active transcriptional chromatin signatures at the type B stage but repressive chromatin signatures at the spermatid stage, whereas *Ttll4*, a spermatid marker, displayed the opposite pattern (Fig. 1b). These datasets provide a thorough genome-wide view of epigenomic landscapes during mouse spermatogenesis to date, insightful for studying this complex developmental process (Fig. 1b–d; Supplementary information, Fig. S2).

### Developmental chromatin signatures during spermatogenesis

To follow epigenomic dynamics during spermatogenesis, similar to ENCODE, we used ChromHMM to define a 15-state model from 7 histone modifications to annotate the genomes throughout spermatogenesis^49,50^ (Fig. 1e; Supplementary information, Fig. S4a, b). We filtered sequences from double-stranded break (DSB) hotspots and Pseudo autosomal Regions (PARs) on the X and Y chromosomes as their regulation is known to be specific to meiotic recombination.^25,46,51^ Each chromatin state was then assigned a descriptive term based on its similarity to known chromatin functions and signatures (as illustrated in Fig. 1e; Supplementary information, Fig. S4b).

Consistent with previous studies,^52^ promoters exhibited the highest accessibility (least nucleosome occupancy) (Fig. 1f; Supplementary information, Fig. S4c, d) and the lowest levels of DNAme (Supplementary information, Fig. S4c, e), followed by enhancers, transcriptional units, and heterochromatin (Fig. 1f; Supplementary information, Fig. S4c–e). Stage-specifically expressed genes, such as *Zbtb16*, *Sohlh1*, *Dmc1*, and *Acr* were deicorated with active marks linked to enhancer, promoter, and transcriptional units at the relevant cell-stages, demonstrating that chromatin states shifted accompanying with stage-specific gene expression (Fig. 1g). The genomic coverage of different chromatin states varies and exhibit dynamic changes throughout spermatogenesis (Fig. 1h; Supplementary information, Fig. S4f, g). On average, about 1.6% of the genome exhibited differences in chromatin states between adjacent developmental stages (mean: 1.6%, 40.3 Mb, range 0.003%–8.4%, 0.07–207.2 Mb) (Supplementary information, Fig. S4f–h and Table S3). We found that late stages of male germ cells displayed broad and elevated H3K4me3 distribution at promoters and their flanking regions (the Pr-F state) (Fig. 1h), discussed further below. Surprisingly, we found that the heterochromatin state (Hc-H3K9me2) exhibited the highest degree of dynamics, followed by enhancer states, across different spermatogenic stages (Fig. 1i; Supplementary information, Fig. S4h). Furthermore, analysis of coordinated dynamics in chromatin signatures associated with male germ cell development revealed that type B spermatogonia, the stage after which mitosis-to-meiosis transition occurs, and mid-pachytene (mP) spermatocytes, the stage during which meiotic recombination and synapsis are completed, underwent the most extensive chromatin state changes (Fig. 1i; Supplementary information, Fig. S4h). In line with this, transcriptomic data showed that mP spermatocytes underwent a transitional stage where spermiogenesis genes are upregulated, while meiotic and mitotic genes are downregulated,^4^ discussed further below. Taken together, these data reveal that major shifts in chromatin states coincide with the expression of stage-specific genes in male germ cell development.

### Two major rearrangements of heterochromatin marks at the mitotic-to-meiotic transition and completion of meiotic recombination and synapsis

We investigated changes in heterochromatin marks, i.e., H3K9me2, H3K9me3, and H3K27me3 and identified two major phases of heterochromatin mark rearrangement that are unique to male germ cell development.

**Fig. 2:**
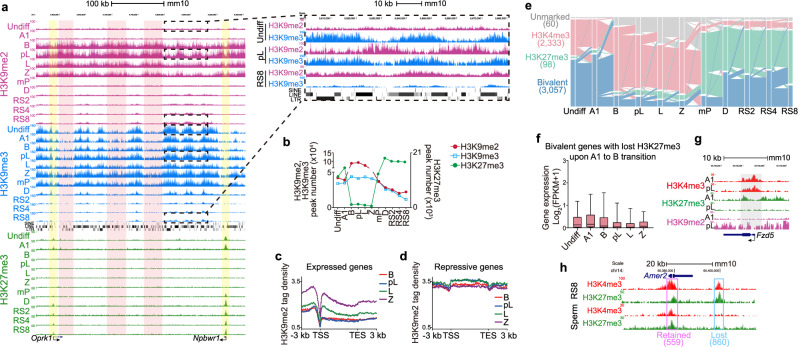
Dynamics of repressive histone marks during mouse spermatogenesis. **a** Snapshots of the UCSC genome browser showing the normalized ChIP-seq read densities of H3K9me2, H3K9me3, and H3K27me3 in a representative genomic region containing *Oprk1* and *Npbwr1* genes. Areas between H3K9me3 peaks are highlighted in light red to indicate increased H3K9me2 enrichment specifically from B to Z stages. Promoter regions, highlighted with yellow shading, are modified by H3K27me3 at various stages, excluding B to Z stages. Right panel shows a magnified view of dashed framed regions for H3K9me2 and H3K9me3 patterns in the indicated stages. SINE, LINE, and LTR elements are shown as black squares. **b** The numbers of H3K9me2, H3K9me3 and H3K27me3 ChIP-seq peaks at 11 stages of mouse spermatogenesis. **c**, **d** Metagene profiles showing the ChIP-seq normalized read densities of H3K9me2 on genes that are expressed (**c**) and repressive (**d**) from B to Z stages. **e** Alluvial plot showing the temporal dynamics of bivalent domains during mouse spermatogenesis. Each line in the plot represents a bivalent gene, and the total regions shown are those classified as bivalent genes in at least one of the analyzed stages. **f** Boxplot illustrating the expression levels of bivalent genes that underwent a loss of H3K27me3 modifications during the transition from A1 to B stages. **g** Snapshots of the UCSC genome browser showing the normalized ChIP-seq read densities of H3K4me3, H3K27me3, and H3K9me2 at A1 and pL stages on a representative bivalent gene, *Fzd5*. **h** Snapshots of the UCSC genome browser showing the normalized ChIP-seq read densities of H3K4me3 and H3K27me3 at RS8 and mature sperm stages over three representative bivalent gene loci, indicating the retained and lost bivalent states during the progression from RS8 stage to mature sperm.

The first rearrangement began in type B spermatogonia and involved a drastic genome-wide increase in H3K9me2 concordant with a global loss of H3K27me3 (Fig. 2a, b; Supplementary information, Fig. S5a). By contrast, H3K9me3 remained relatively constant during these stages (Fig. 2a, b). Furthermore, the newly established H3K9me2 peaks did not overlap with H3K9me3 peaks, which were enriched over LINE and LTR but not SINE elements (Fig. 2a; Supplementary information, Fig. S5b, c). ChIP-qPCR analyses confirmed that stronger H3K9me2 signals were detected in the B and Z stages at the tested loci (Supplementary information, Fig. S5d). It is known that male germ cells, from type B spermatogonia to zygotene spermatocytes, express lower number of transcripts compared to other stages during spermatogenesis and exhibit global repression of transcription (Supplementary information, Fig. S5e).^53–55^ Yet the link to histone modifications remains incompletely understood. Here, we found that meiotic H3K9me2 was highly enriched on repressive but relatively less over active genes (Fig. 2c, d), suggesting that this modification, perhaps together with H3K9me3, might be involved in meiotic gene repression,^56^ a matter for future investigation.

The second rearrangement occurred at the mP stage, where meiotic recombination and synapsis are completed. We observed global loss of H3K9me2 and H3K9me3 at the mP stage, coinciding with robust transcriptional activation (Fig. 2a, b; Supplementary information, Fig. S5e).^4^ Consistent with this loss, H3K9me2 demethylase *Kdm3*-deficient spermatids were associated with downregulation of several key genes essential for spermiogenesis, including *Crem*, *Tnp1/2*, and *Prm1/2*.^16,57,58^ Of note, after the global removal, the remaining H3K9me3 and H3K9me2 marks were left predominantly at LINEs and LTRs, highlighting their roles in retrotransposon silencing during the late stages of male germ cell development (Fig. 2a; Supplementary information, Fig. S5f). Interestingly, in contrast to the erasure of H3K9me2/3, H3K27me3 levels were quickly reestablished at the mP stage (Fig. 2a, b; Supplementary information, Fig. S5g, h). Re-established H3K27me3 predominantly marked mitotic/meiotic expressed genes that were covered by H3K4me3 before the mP stage, but need to be silenced after the mP stage^59^ (i.e., *Drmt1/3, Sohlh1/2, Stra8, Rarg, M1ap*) (Supplementary information, Fig. S5g–i and Table S4). This suggests the crucial role of H3K27me3 in maintaining gene repression during spermiogenesis. However, H3K27me3 was depleted from LINE and LTRs, which were mainly covered by H3K9me2/3 in the late stages of male germ cells as described above (Supplementary information, Fig. S5c). Of note, both stage-specific genome-wide switches in repressive marks were associated with expression changes in the relevant chromatin modifying enzymes, EHMT/G9A,^56^ SUV39H1/2,^60^ EZH1/2,^19,61^ KDM6s,^62,63^ KDM4D^64,65^ and KDM3A^16^ (Supplementary information, Fig. S5j, k).

In addition, we discovered that most H3K4me3-H3K27me3 bivalent genes, which were originally thought to remain in a bivalent state throughout spermatogenesis,^21,22,66^ lost their bivalency due to the loss of H3K27me3 from the B spermatogonia to zygotene spermatocytes (Fig. 2e; Supplementary information, Fig. S6a, b and Table S4). However, these genes did not exhibit transcriptional de-repression in these stages, likely due to the repressive effects of H3K9me2 (Fig. 2f, g; Supplementary information, Fig. S6c). Starting from the mP stage, H3K27me3 was re-established, leading to the formation of numerous de novo H3K4me3-K27me3 bivalent domains,^21^ with 39.4% persisting as bivalent and 60.6% becoming H3K27me3-only regions in mature sperm (Fig. 2e, h; Supplementary information, Fig. S6d).

Taken together, these findings reveal two major rearrangements of heterochromatin markers that are coincident with previously reported events such as meiotic transcription repression, postmeiotic global transcription activation, postmeiotic silencing of mitotic/meiotic genes, and silencing of retrotransposons.

### Establishment of conserved broad and robust H3K4me3 and H3K27ac domains in spermatids

Next, we investigated changes in active histone marks (mentioned above in Fig. 1h, i.e., Pr-F state) during spermatogenesis. Notably, we found that H3K4me3 and H3K27ac underwent more significant global changes compared to H3K4me1 and H3K36me3 across male germ cell development (Fig. 1c, d; Supplementary information, Fig. S2i, j). Two significant waves of H3K4me3 elevation occurred at the leptotene/zygotene and round spermatid (RS) stages, respectively (Fig. 1c, d; Supplementary information, Fig. S2i, j). The first wave, coupled to PRDM9 activation and the formation of DSB hotspots, was reported previously.^25,46^ However, the second wave of H3K4me3 elevation, concurrent with high levels of the forementioned Pr-F state (Fig. 1h), has not been previously reported.

**Fig. 3:**
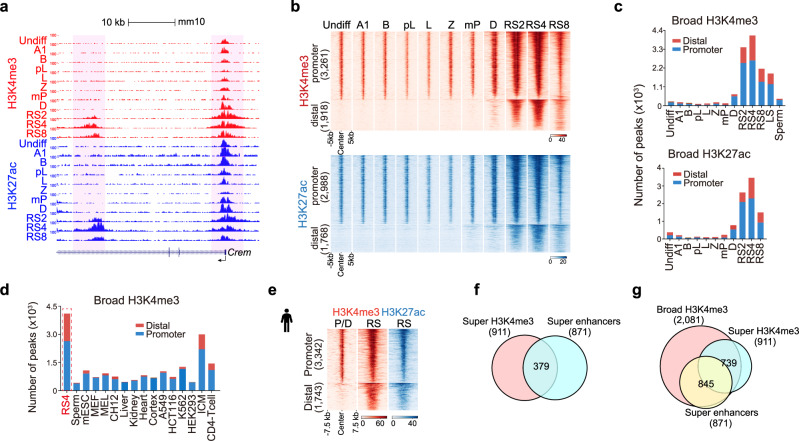
Dynamic changes in broad H3K4me3 and H3K27ac domains during spermiogenesis. **a** Snapshots of the UCSC genome browser showing the normalized ChIP-seq read densities of H3K4me3 (red) and H3K27ac (blue) at the *Crem* gene locus during mouse spermatogenesis. Regions shaded in pink highlight two broad H3K4me3 and H3K27ac peaks at the distal (left) and promoter (right) regions of the *Crem* gene, emphasizing the broad enrichment pattern of H3K4me3 and H3K27ac at the RS stages. **b** Heatmaps showing the normalized ChIP-seq read densities of H3K4me3 (red, upper panel) or H3K27ac (blue, lower panel) during mouse spermatogenesis on all broad H3K4me3 or H3K27ac peaks identified at the RS stages. **c** Stacked chart showing the numbers of broad H3K4me3 peaks (upper panel) from undifferentiated spermatogonia (Undiff) to mature sperm and H3K27ac peaks (lower panel) from Undiff to RS8 on promoter and distal regions. The broad peaks are defined as ChIP-seq peaks exceeding 5 kb in length. The promoter regions are defined as ± 2 kb from the TSS, and the distal regions are defined as the genomic regions beyond ± 2 kb from the TSS. LS denotes elongating spermatid steps 10–12, and sperm denotes the mature sperm stage. **d** Stacked chart showing the number of broad H3K4me3 peaks in RS4 compared to a range of indicated cell types. These include diverse mouse and human cell lines, CD4^+^ T cells, and early embryonic cells. **e** Heatmaps showing the normalized ChIP-seq read densities of H3K4me3 (red, left panel) and H3K27ac (blue, right panel) in human pachytene/diplotene (P/D) and/or RS. The visualization encompasses all broad H3K4me3 or H3K27ac peaks identified in human RSs. **f** Venn diagrams showing the overlap between H3K4me3-based SEs (super H3K4me3) and H3K27ac-based SEs identified by ROSE in mouse RSs. **g** Venn diagrams showing the overlap among H3K4me3-based SEs, H3K27ac-based SEs and broad H3K4me3 peaks.

Three key patterns were observed for the second H3K4me3 wave in spermatids. Firstly, up to 5136 promoters and enhancers were modified by these newly established exceptionally broad and strong H3K4me3 domains (with each over 5 kb, on average 8.7 kb). These domains are hereafter called spermatid broad H3K4me3, and 65.7% of them coincided with more than 76% of broad H3K27ac peaks (Fig. 3a–c; Supplementary information, Fig. S7a, b and Table S5). This contrasts with regular H3K4me3 signals which are typically found as narrow peaks (∼1–2 kb) at the transcription start sites (TSSs).^29^ More recently, the term “broad H3K4me3” was used to characterize relatively extensive H3K4me3 domains in inner cell mass (ICM) and in human CD4^+^ T cells.^38,40^ However, the number and strength of broad H3K4me3 domains in spermatids drastically surpassed those in other cell contexts (Fig. 3d; Supplementary information, Fig. S7c–e).^38,40^ Secondly, spermatid broad H3K4me3 domains displayed dynamic changes during spermiogenesis (Fig. 3b, c; Supplementary information, Fig. S7f, g). We observed that the spermatid broad H3K4me3 signals were first detected in diplotene spermatocytes and increased during RS2 (3407), reaching their peak during RS4 (4090). These broad domains decreased during RS8 (2158) and LS (steps 10–12 elongating spermatids) (1840), with relatively few left in the mature sperm (407) (a total of 5136 cross all these stages, Fig. 3a–c; Supplementary information, Fig. S7g). Intriguingly, broad H3K27ac exhibited a similar pattern to spermatid broad H3K4me3 (Fig. 3a–c; Supplementary information, Fig. S7a, b, f, g). Lastly, spermatid broad H3K4me3 domains were not only deposited at TSSs (3055 out of 5136 peaks, 59.5%) but also found at thousands of distal enhancer regions (2081 out of 5136 peaks, 40.5%) (Fig. 3b, c). In comparison, around 60% of regular H3K4me3 was distributed in distal enhancer regions (Supplementary information, Fig. S7h). Importantly, we also found that the broad H3K4me3 and H3K27ac domains are conserved in human spermatids (Fig. 3e; Supplementary information, Fig. S7i–k).

### Spermatid broad H3K4me3 domains display SE features

We and others have previously reported H3K4me3-marked enhancers that are negatively regulated by RACK7/KDM5C, presenting an overly activated state.^67–69^ However, enhancer-associated H3K4me3 is generally much weaker than promoter H3K4me3 in most reported systems (Supplementary information, Fig. S7d).^38,40^ Additionally, the number of distal broad H3K4me3 domains detected in those reported models was relatively low, on average less than 100 (Fig. 3d).^38^ Here, we found that the spermatid broad H3K4me3 domains, regardless of association with TSSs (59.5%) or distal regions (40.5%), exhibited similar levels of signal strength (Supplementary information, Fig. S7d). In mouse spermatogenesis, H3K27ac-based enhancers and SEs have been characterized previously.^23,70^ However, the feature of H3K4me3 in enhancer regions remain unexplored. To address this, we applied the ROSE program^71,72^ to our H3K4me3 ChIP-seq data from RSs (RS2, RS4 and RS8), and identified 911 merged “super” H3K4me3 domains. Comparative analysis revealed that 41.6% (379 out of 911) of these super H3K4me3 domains overlapped with SEs (Fig. 3f). Further analysis showed that the majority of “super” H3K4me3 domains (739 out of 911) and SEs (845 out of 871) overlap with broad H3K4me3 domains, highlighting these regions as some of the most active enhancers in RSs (Fig. 3g).

### Spermatid broad H3K4me3 domains are associated with highly expressed genes linked to spermatid identity

**Fig. 4:**
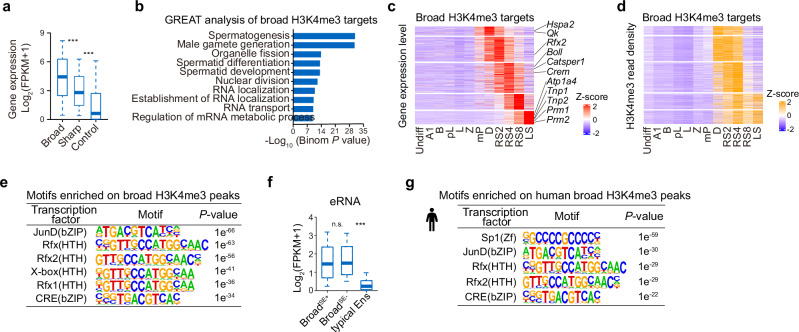
Spermatid-specific broad H3K4me3 domains are coupled to transcriptional potentials of key genes for spermiogenesis. **a** Boxplot showing the expression levels of target genes associated with broad, sharp, and control H3K4me3 peaks at the RS4 stage of mouse spermatogenesis. ***P < 0.001. **b** Functional enrichment analysis of broad H3K4me3 marked genes in mouse spermatids utilizing GREAT to infer potential functional pathways and associated biological processes. **c**, **d** Heatmaps showing the RNA expression levels (**c**) and broad H3K4me3 intensities (**d**) of RS broad H3K4me3-marked genes throughout mouse spermatogenesis. Genes are sorted based on the peak expression timing, progressing from early to late stages. **e** Transcription factor-binding motifs enriched within broad H3K4me3 domains in mouse RSs. **f** Boxplots showing the expression levels of enhancer RNAs in RSs across three categories: broad H3K4me3 domains that overlap with SEs (Broad^SE+^), broad H3K4me3 domains without overlapping with SEs (Broad^SE–^), and typical enhancers (Ens). **g** Transcription factor-binding motifs enriched within broad H3K4me3 domains in human RSs.

Given that spermatid broad H3K4me3 domains significantly overlapped with SEs and showed even broader genomic coverage, we hypothesized that they might play a role in transcriptional activation during spermatid development. To investigate this, we analyzed the transcriptional levels of genes marked by spermatid broad H3K4me3 domains, sharp H3K4me3 domains (top 500 regular H3K4me3 domains with the highest density), and control peaks (500 randomly selected regular H3K4me3 domains) in spermatids. We found that genes marked by spermatid broad H3K4me3 produced significantly higher transcript levels than genes associated with regular or control H3K4me3 domains (Fig. 4a). Notably, broad H3K4me3-linked genes were enriched for key functions integral to spermatid development (Fig. 4b) and exhibited a temporal expression that mirrored the dynamics of broad H3K4me3 (Fig. 4c, d). Furthermore, the strength of broad H3K4me3 correlated with the expression levels of associated genes, such as *Crem* and *Prm1*, which emerge as the most highly expressed genes during the relevant stages of spermatid development^8,73^ (Figs. 3a, 4c, d; Supplementary information, Fig. S8a, b). Additionally, motif enrichment analyses revealed that transcription factor motifs (RFX2 and CREM) linked to spermatid development^6–8^ were enriched within spermatid broad H3K4me3 domains (Fig. 4e). Supporting the notion that strong enhancer activity is associated with spermatid broad H3K4me3 domains, we found elevated transcription of enhancer RNAs (eRNAs) within these domains, indicative of heightened enhancer activities, relative to regular H3K4me3 enhancers (Fig. 4f).

Importantly, we also observed higher transcriptional levels at genes marked by broad H3K4me3 domains in human spermatids (Supplementary information, Fig. S8c).^74^ These genes showed functional enrichment of spermiogenesis by gene ontology (GO) term analysis and were enriched for similar transcription factor motifs like RFX2 and CREM as mouse broad H3K4me3 genes (Fig. 4g; Supplementary information, Fig. S8d). In conclusion, our findings demonstrate that spermatid broad H3K4me3 domains are linked to key spermiogenesis genes, exhibiting robust transcriptional output and stage-specific expression patterns in both mice and humans.

### SETD1B methyltransferase is required for the formation of spermatid broad H3K4me3 domains

**Fig. 5:**
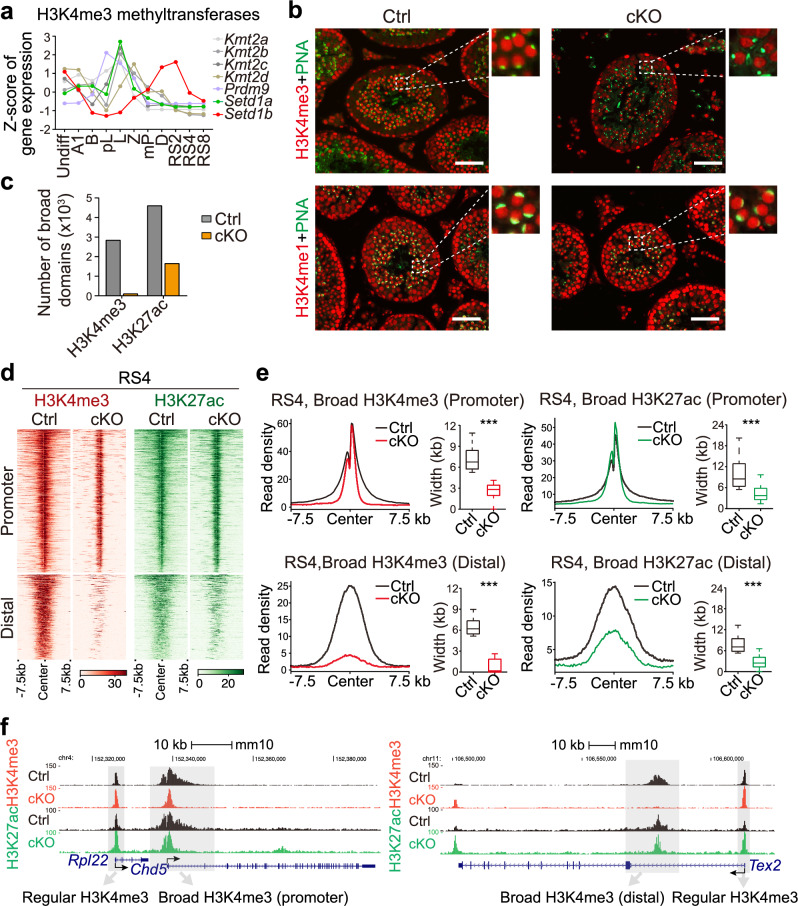
SETD1B is responsible for the establishment of broad H3K4me3 domain during spermiogenesis. **a** Line chart showing the gene expression profiles of seven H3K4me3 methyltransferases across 11 stages of mouse spermatogenesis, determined by RNA-seq. **b** Immunofluorescent (IF) staining for H3K4me3 (red, upper panel) or H3K4me1 (red, lower panel) in sections of adult control and *Setd1b* cKO testes. Acrosome marker peanut lectin (PNA, green) was co-stained to determine the specific stages of the seminiferous epithelium. Magnified views of dashed framed regions were shown on the right side of each image. Scale bars, 100 μm. **c** Bar plot showing the numbers of broad H3K4me3 and broad H3K27ac domains identified at the RS4 stage of RSs in control and *Setd1b* cKO mice. **d** Heatmaps showing the normalized read densities of H3K4me3 (red, left panel) and H3K27ac (green, right panel) across all broad H3K4me3 and H3K27ac domains identified at the RS4 stage of RSs in both control and *Setd1b* cKO mice. Broad domains are categorized into promoter and distal groups based on their genomic locations. **e** Metagene profile plots showing the averaged ChIP-seq read densities of H3K4me3 and H3K27ac on promoter (upper) and distal (lower) broad H3K4me3 (left) and H3K27ac (right) peaks identified at the RS4 stage of RSs in both control and *Setd1b* cKO mice. Box plots in the right panel show the width of peaks. **f** Snapshot of the UCSC genome browser showing the normalized ChIP-seq read densities of H3K4me3 and H3K27ac at the *Rpl22* locus (regular H3K4me3 peak), *Chd5* locus (promoter broad H3K4me3 peak), and *Tdrd7* locus (regular and distal broad H3K4me3 peaks) at the RS4 stage in control and *Setd1b* cKO mice. Broad and regular H3K4me3 peaks were shaded in grey color.

We next tested which H3K4 methyltransferase(s) generates spermatid broad H3K4me3 and assessed the impact of perturbing these domains on the spermatid transcriptome. Six KMT2 methyltransferases and PRDM9 are known to catalyze H3K4me3 formation. Of these, only *Setd1b* expression coincided with the formation of spermatid broad H3K4me3 domains, as revealed by RNA-seq (Fig. 5a). This alignment is further supported by single-cell RNA-seq data (Supplementary information, Fig. S9a).^4^ To confirm the role of *Setd1b* in the formation of spermatid broad H3K4me3 domains, we generated a *Setd1b*-floxed mouse line using CRISPR/Cas9 and specifically inactivated *Setd1b* in male germ cells from type A1 spermatogonia onwards using a *Stra8*-GFP Cre line (hereafter referred to as *Setd1b* cKO) (Supplementary information, Fig. S9b).^75^ RNA-seq results demonstrated that exon 5 of *Setd1b* transcripts was absent, which is also verified by genotyping PCR, revealing high knockout (KO) efficiency (Supplementary information, Fig. S9c, d). Immunostaining analyses showed that, in *Setd1b* cKO testes, spermatid H3K4me3 but not H3K4me1 signals were greatly reduced, suggesting that *Setd1b* is required for generation of RS broad H3K4me3 (Fig. 5b). ChIP-seq analyses demonstrated that broad H3K4me3 domains were completely lost in *Setd1b* cKO spermatids, while regular H3K4me3 signals remained unaffected (Fig. 5c–f; Supplementary information, Fig. S9e). More specifically, the H3K4me3 signals at distal enhancers largely vanished, while promoter broad H3K4me3 regions were also lost but leaving regular H3K4me3 domains (Fig. 5d–f), likely catalyzed by other methyltransferase(s). We also found that H3K27ac was greatly reduced at the broad H3K4me3 enhancers but largely unaffected at the broad H3K4me3 promoters in *Setd1b* cKO spermatids, implying that distal enhancer broad H3K27ac is more relied on SETD1B compared to promoter H3K27ac (Fig. 5c–f). Collectively, these results demonstrate that SETD1B is the major methyltransferase responsible for generating spermatid broad H3K4me3 domains.

### SETD1B regulates Pol II distribution between broad and regular H3K4me3 regions and activates genes required for spermatid development

**Fig. 6:**
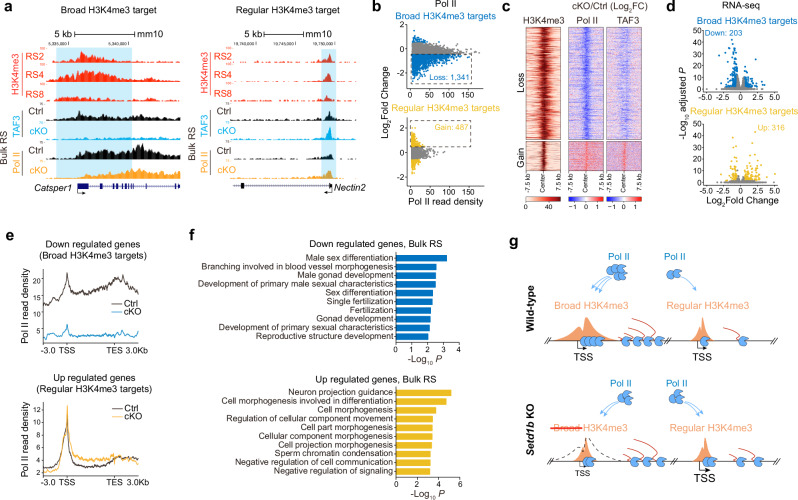
SETD1B deficiency leads to Pol II redistribution and transcriptional dysregulation. **a** Snapshots of the UCSC genome browser showing the normalized ChIP-seq read densities of H3K4me3 across various stages of RSs, along with TAF3 and RNA Pol II read densities from wild-type control and *Setd1b* cKO bulk RS samples, over the *Catsper1* (a broad H3K4me3 target) and *Nectin2* (a regular H3K4me3 target) gene loci. Blue shading highlights regions with broad H3K4me3 (left panel) and regular H3K4me3 (right panel) peaks. **b** MA plots showing changes in RNA Pol II enrichment at the promoter regions of broad H3K4me3 (upper panel) or regular H3K4me3 (lower panel) marked genes following *Setd1b* depletion. The numbers of broad H3K4me3 targets with decreased Pol II occupancy (loss) and regular H3K4me3 targets with increased Pol II occupancy (gain) are indicated. **c** Heatmap showing changes in RNA Pol II and TAF3 enrichment on 1341 broad H3K4me3 marked genes exhibiting decreased Pol II occupancy (loss) and 487 regular H3K4me3 marked genes displaying increased Pol II occupancy (gain). The corresponding H3K4me3 enrichment pattern in those regions in wild-type RSs is depicted on the left. **d** Volcano plot showing differential gene expression between control and *Setd1b* cKO bulk RSs for broad H3K4me3-marked genes (upper panel) and regular H3K4me3-marked genes (lower panel). Significant differential expression was defined by a threshold of a Log_2_ FC ≥ 0.5 or ≤ –0.5, coupled with an adjusted *P* < 0.05. **e** Metagene profile plots showing the normalized ChIP-seq read densities of RNA Pol II for down-regulated broad H3K4me3-marked genes and up-regulated regular H3K4me3-marked genes when comparing *Setd1b* cKO and control bulk RS. **f** GO analysis for down- and up-regulated genes in bulk RSs when comparing *Setd1b*cKO and control mice. **g** Model of SETD1B-mediated broad H3K4me3 regulating gene expression through promoting Pol II occupancy.

A link between H3K4me3 and gene activation, in particular molecularly through TAF3–H3K4me3 interaction and PIC assembly, has previously been established.^35^ H3K4me3 was also recently shown to regulate Pol II occupancy and transcription in mouse embryonic stem cells using an acute degradation system that disrupts all KMT2 functions.^33,34^ We therefore tested whether SETD1B-mediated broad H3K4me3 domains influence transcription and Pol II occupancy in the developing spermatids. We first performed TAF3 and Pol II (N-terminal domain) ChIP-seq in mixed populations of RSs (bulk RS) from control and *Setd1b* cKO mice. In the wild-type mice, we found significantly higher levels of promoter-bound and elongating Pol II, as well as TAF3 occupancy, over genes marked by broad H3K4me3 at TSSs compared to genes associated with sharp and control H3K4me3 (Supplementary information, Fig. S10a). Pol II and TAF3 occupancies at distal broad H3K4me3 domains showed the same trend (Supplementary information, Fig. S10b). Furthermore, we observed a decrease in nucleosome occupancy at promoters marked by broad H3K4me3 decreases as spermatogenic cells developed to the RS stage (Supplementary information, Fig. S10c). We also identified DNA hypomethylation at 50% of SETD1B-mediated H3K4me3 domains (defined as those with an average DNAme level below 0.3) (Supplementary information, Fig. S10d). These findings are consistent with our observation that spermatid broad H3K4me3 domains are associated with highly expressed genes. Importantly, we found that *Setd1b* depletion caused a strong reduction in TAF3 and Pol II occupancy over broad H3K4me3 covered TSSs, gene bodies, and enhancers at a total of 1341 sites, demonstrating that the broad H3K4me3 domains are key for PIC binding (Fig. 6a–c; Supplementary information, Fig. S10e–g). The most drastic reduction was observed at distal enhancers, consistent with the higher SETD1B dependence of broad H3K4me3 and H3K27ac in the distal genomic regions (Supplementary information, Fig. S10f, g). By contrast, we observed an increase in TAF3 and Pol II occupancy at 487 of the regular H3K4me3 sites, indicating a redistribution of Pol II from broad H3K4me3 regions to regular H3K4me3 sites upon *Setd1b* depletion (Fig. 6a–c). This trend was consistent with our RNA-seq data where 203 broad H3K4me3 target genes exhibited decreased expression levels. By contrast, 316 regular H3K4me3 target genes showed increased transcript levels ( | Log_2_FC | > 0.5, *P*_adj_ < 0.05) in bulk RS upon *Setd1b* loss (Fig. 6d; Supplementary information, Table S6). Comparing Pol II occupancy between down-regulated broad H3K4me3 genes and up-regulated regular H3K4me3-associated genes further confirmed that Pol II occupancy shifted to regular H3K4me3 upon *Setd1b* depletion (Fig. 6e). GO analyses revealed that the down-regulated broad H3K4me3 genes were enriched in functions related to spermatid development, whereas the up-regulated genes were predominantly linked to morphogenesis-associated functions (Fig. 6f).

### SETD1B-mediated broad H3K4me3 controls stage-specific temporal patterns of gene expression

**Fig. 7:**
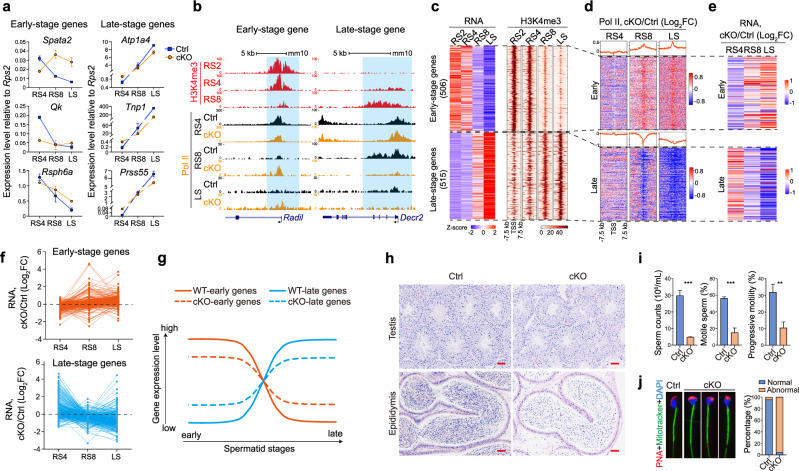
SETD1B-mediated broad H3K4me3 ensures accurate expression timing of stage-specific genes and spermatid development. **a** Line plots depicting the expression dynamics of representative early-stage genes (left panel) and late-stage genes (right panel) marked by broad H3K4me3, observed across RS4, RS8, and LS stages in both control and *Setd1b* cKO mice. **b** Snapshots of the UCSC genome browser showing the normalized ChIP-seq read densities of H3K4me3 across various spermatid stages from wild-type mice, along with normalized RNA Pol II read density in RS4, RS8 and LS stages from wild-type control and *Setd1b* cKO mice, over the *Radil* (an early-stage broad H3K4me3 target) and *Decr2* (a late-stage regular H3K4me3 target) gene loci. Blue shading highlights the H3K4me3-covered genomic regions. **c** Classification of broad H3K4me3 target genes based on their peak expression timing: early (RS2 & RS4) vs late (RS8 & LS10) stages (left panel). Corresponding H3K4me3 read densities for early-stage and late-stage genes are displayed in the right panel. **d** Heatmap showing the variation in RNA Pol II enrichment on early-stage and late-stage broad H3K4me3 marked genes across RS4, RS8 and LS10 stages. **e** Heatmap showing the variation in gene expression on early-stage and late-stage broad H3K4me3-marked genes across RS4, RS8 and LS10 stages. **f** Multiple line charts showing the variation in gene expression on early-stage and late-stage broad H3K4me3-marked genes across RS4, RS8 and LS10 stages. **g** Models of SETD1B-mediated broad H3K4me3 in regulating stage-specific temporal gene expression during spermiogenesis. **h** H&E staining of wild-type control and *Setd1b* cKO testes (upper panel) or epididymis (lower panel) sections from 8-week-old mice. Scale bars, 100 µm. **i** Number, total, and progressive motility of caudal epididymal sperm from adult control and *Setd1b* cKO mice. Data are presented as mean ± SD, ***P < 0.001, **P < 0.01, Student’s *t*-test (*n* = 3). **j** Fluorescence staining of caudal epididymal sperm from control and *Setd1b* cKO mutant with fluorescence dye-labeled peanut lectin (PNA, red) for acrosome, MitoTracker Green FM (green) for mitochondria, and DAPI (blue), respectively (left panel). Stacked chart showing the proportion of sperm with normal or abnormal head morphology from control and *Setd1b* cKO mice (right panel).

The bulk RS data underscore the crucial role of SETD1B-mediated broad H3K4me3 domains in promoting PIC binding and gene activation. However, changes to the number of genes comprising the transcriptome were less pronounced upon *Setd1b* loss than in recent studies examining H3K4me3 regulation of Pol II occupancy and transcription.^33,34^ Given that broad H3K4me3-associated genes display distinct temporal expression patterns during spermatid development, we pursued a further investigation of SETD1B-dependent stage-specific effects. We isolated synchronized populations of spermatids and analyzed differentially expressed genes in RS4 and LS (steps 10–12 elongating spermatids) upon *Setd1b* depletion (Supplementary information, Fig. S11a). RS4 cells have the highest broad H3K4me3 levels, while LS represents a later developmental stage with fewer broad H3K4me3 domains. Consistent with the idea that H3K4me3 promotes transcription, *Setd1b* KO led to the downregulation of 647 genes (including 341 broad H3K4me3 genes) in RS4 and 1125 genes (including 741 broad H3K4me3 genes) in LS (Supplementary information, Fig. S11b and Table S7). Notably, many down-regulated genes were crucial players in spermiogenesis, including *Boll, Usp2, Hspa2, Qk*, and *Brwd1* in *Setd1b*-deficient RS4 cells, and *Tnp1, Tnp2, Prm1, Prm2* and *Spata19* in *Setd1b*-deficient LS (Supplementary information, Fig. S11b, c). However, to our surprise, we again observed that *Setd1b* KO led to upregulation of 874 genes (including 321 broad H3K4me3 genes) in RS4 and 334 genes (including 108 broad H3K4me3 genes) in LS (Supplementary information, Fig. S11b, c). Further examination of the up-regulated broad H3K4me3 genes found that they are generally highly expressed at other stages. For instance, *Spata2*, *Qk*, *Rsph6a* and *Radil*, which are most highly expressed in the RS4 population, showed elevated expression in LS upon *Setd1b* depletion (Fig. 7a, b). Similarly, *Atp1a4, Tnp1*, *Prss55* and *Decr2* are prominently expressed during LS and were upregulated in RS4 upon *Setd1b* depletion (Fig. 7a, b). These findings indicate that SETD1B not only promotes transcriptional output but also dictates the accurate expression timing of stage-specific genes.

The above findings indicated that SETD1B-mediated broad H3K4me3 domains serve as landmarks for enhanced Pol II recruitment. The absence of SETD1B in *Setd1b* KO spermatids leaves only regular H3K4me3-marked promoters, which is insufficient in allocating the proper amount of Pol II to stage-specific genes, ultimately compromising transcription timing. To support this theory, we then switched the focus of our analysis to gene expression timing. Using RNA-seq data we filtered out 1021 stage-specifically expressed broad H3K4me3 genes and categorized them into two groups based on the timing of their peak expression: early spermiogenesis genes (highest expression in RS2 and RS4) and late spermiogenesis genes (highest expression in RS8 and LS) (Fig. 7c; Supplementary information, Table S8). Notably, the breadth and strength of their H3K4me3 levels in each stage were well-correlated with their temporal expression patterns (Fig. 7c). Importantly, the loss of *Setd1b*-mediated broad H3K4me3 caused changes in Pol II distribution (Fig. 7d; Supplementary information, Fig. S11d) with two major outcomes: 1) reduced expression of these genes at the stages when they should achieve maximal expression; 2) increased expression of these genes at stages where they would normally exhibit lower expression levels (Fig. 7e, f). Despite this increased expression upon *Setd1b* loss, the levels remained lower than their normal peak expression levels, further confirming that broad H3K4me3 promotes gene transcription by facilitating Pol II binding (Fig. 7a; Supplementary information, Fig. S11e). The second consequence also caused comprised expression timing of stage-specific genes, resulting in the phenomenon of “early gene up late and late gene up early” in *Setd1b*-deficient spermatids (Fig. 7e–g), consistent with our hypothesis.

*Setd1b* depletion also had a more pronounced impact on transcriptional changes in LS compared to RS4 (Fig. 7e; Supplementary information, Fig. S11b). Notably, we found that as spermatid development progressed, there was a significant decline in the quantities of PIC components such as Pol II and TAF3 (Supplementary information, Fig. S11f–h). These results suggest that the divergent transcriptional effects of *Setd1b* depletion between the early and late spermatid stages might stem from a substantial decrease in Pol II levels and PIC components during the later stages of spermiogenesis.

Collectively, our findings reveal that *Setd1b*-mediated broad H3K4me3 plays a pivotal role in Pol II occupancy and transcription. The loss of broad H3K4me3 triggers an aberrant redistribution of Pol II from broad to regular H3K4me3 sites (Fig. 6g), leading to an impaired output and wrecked timing of stage-specific gene expression which are critical for spermatid development (Fig. 7g). However, the extent of such effects varies across different stages, with a more prominent effect observed in late RS stages characterized by limited PIC quantities.

### RFX2 interacts with SETD1B to establish broad H3K4me3 during spermiogenesis

Transcription factors play a crucial role in initiating chromatin modification by recruiting histone-modifying enzymes, thereby regulating gene expression. The aforementioned motif analysis of broad H3K4me3 sites has revealed enrichment in binding sequences for transcription factors such as the *Rfx* family and *Crem* during spermiogenesis (Fig. 4e). Among these factors, RFX2, known for its role in cilia development, is essential for spermiogenesis.^5,7^ Importantly, *Setd1b* exhibits a similar expression pattern to *Rfx2* during spermatid development.^76,77^ Moreover, ChIP-seq data for RFX2 showed an overlap with broad H3K4me3 regions (Supplementary information, Fig. S12a). Thus, it is tempting to speculate that RFX2 might be involved in the broad H3K4me3 establishment by SETD1B, thereby mediating the formation of broad H3K4me3 modifications at key genes involved in spermiogenesis. Since *Rfx2* loss caused developmental arrest at RS2,^7^ we collected RSs at RS2 from both control and *Rfx2* KO mice. ChIP-seq analysis revealed a significant reduction in approximately 74.7% (1620 out of 2169 peaks) of the broad H3K4me3 domains, including those at promoters and distal enhancers, in *Rfx2* KO spermatids (Supplementary information, Fig. S12b–d). Additionally, 43.9% (953 out of 2169 peaks) of the broad H3K4me3 domains were converted to regular H3K4me3 domains (Supplementary information, Fig. S12e, f), indicating at least partial dependence of broad H3K4me3 formation on RFX2. To explore the potential interaction between RFX2 and SETD1B, we overexpressed 2× Flag-tagged RFX2 and HA-tagged SETD1B in HEK 293T cells. Co-immunoprecipitation (Co-IP) experiments confirmed an interaction between RFX2 and SETD1B (Supplementary information, Fig. S12g). These findings suggest that RFX2 could recruit SETD1B to establish the specificity of broad H3K4me3 modifications. Future studies should investigate compensatory mechanisms involving other transcription factors to fully elucidate the regulation of SETD1B-mediated broad H3K4me3 modifications.

### Disruption of SETD1B and broad H3K4me3 results in male infertility and aberrant spermiogenesis

We next examined the phenotypes resulting from germ cell-specific inactivation of *Setd1b* and its function on broad H3K4me3 formation. We found that male mice lacking *Setd1b* in germ cells (*Setd1b* cKO) were infertile, and testes from *Setd1b* cKO mutants were markedly smaller than those from control littermates (Supplementary information, Fig. S12h). Hematoxylin and Eosin (H&E) staining showed less elongated spermatids in *Setd1b* cKO testes and fewer sperms in *Setd1b* cKO epididymis compared to controls (Fig. 7h). However, *Setd1b* cKO seminiferous tubules exhibited no detectable abnormalities during meiosis and mitosis (Fig. 7h). We further found that the number and motility of caudal epididymal sperm in the *Setd1b* cKO mutants were significantly lower than those in the controls by computer-assisted sperm analysis (CASA) (Fig. 7i). The majority (>  95%) of mature sperm from *Setd1b* cKO mutants had abnormal heads (Fig. 7j). Sperm morphology analysis using transmission electron microscope (TEM) revealed that chromatin was less condensed in *Setd1b* cKO mutants (Supplementary information, Fig. S12i). Collectively, all these documented defects in spermatid development within *Setd1b* cKO mice aligned with the perturbation of gene expression of *Setd1b*-deficient spermatids. These findings reveal that SETD1B-mediated broad H3K4me3 plays crucial roles in male fertility and proper spermiogenesis by controlling stage-specific gene expression in spermatids.

## Discussion

We have previously developed an approach to isolate homogeneous male germ cells at defined substages from the testes, enabling us to successfully dissect the transcriptional regulation during spermatogenesis.^4^ In this study, we systematically generated epigenomes of homogeneous male germ cells spanning key developmental stages of spermatogenesis. Our findings reveal that chromatin states, particularly heterochromatin and enhancer states, undergo profound and dynamic changes throughout spermatogenesis. Notably, these changes predominantly occur during two critical stages — in type B spermatogonia and mP — and coincide with the temporal silencing and activation of genes during spermatogenesis.

Additionally, we have identified two significant rearrangements of heterochromatin marks — including H3K9me2 and H3K27me3 — that occur in a mutually exclusive manner. We suggest that these rearrangements may orchestrate the timely silencing of transcription during these specific stages, and create a permissive chromatin environment for gene activation in subsequent stages of spermatogenesis.

Notably, we uncovered exceptionally broad (with each > 5 kb) and robust H3K4me3 domains associated with highly expressed genes in both mouse and human spermatids. These domains often coincide with H3K27ac and have features of SEs, highlighting a novel feature of H3K27ac-defined enhancers in RSs. Importantly, spermatid broad H3K4me3 regions differ from previously reported broad H3K4me3 domains in oocytes, ICM cells, CD4^+^ T cells, and tumor cells, in terms of their breadth and intensity (Fig. 3d; Supplementary information, Fig. S7c–e).^38–41^ Moreover, we demonstrate that SETD1B/KMT2G, an understudied KMT2, is the primary methyltransferase that establishes these broad H3K4me3 domains in spermatids. We found that, in the absence of *Setd1b*, these broad domains were converted into regular H3K4me3 domains at promoters, while being almost eliminated from distal enhancer regions. These findings indicate that redundant KMT2(s) might catalyze and maintain the regular H3K4me3 at TSSs but not at enhancers in spermatids. In oogenesis, loss of *Setd1b* function leads to redistribution of H3K4me3 and impairs oogenesis.^78,79^ Recent studies have suggested that *Setd1b* plays a role in modulating the broadness of H3K4me3 domains, in mouse embryonic stem cells and neuronal tissues, while the broadness was not clearly defined.^42,43^ Compared with these studies, our study has unveiled a more specific role and a more robust influence of *Setd1b* on broad H3K4me3 in spermatids. Crucially, SETD1B-mediated broad H3K4me3 domains are more potent in recruiting Pol II compared to regular H3K4me3 regions in male germ cells. We found that RFX2, an essential transcriptional factor for spermatid development, is able to bind SETD1B, and is required for the establishment of broad H3K4me3 domains. Inactivation of *Setd1b* resulted in Pol II redistribution from broad H3K4me3 domains to regular H3K4me3 regions, consequently, disrupting the expression output and timing of genes essential for spermatid development, leading to impaired spermiogenesis and consequent male infertility. Such dependence of Pol II distribution and gene expression time on broad H3K4me3 has been unrecognized previously, largely due to inability in dissecting the non-redundant role of each H3K4me3 methyltransferase in other systems (see below). Of note, genetic mutations in *SETD1B* are associated with neurodevelopmental disorders^80,81^ and cancers.^82,83^ Our work reveals a previously unrecognized role of SETD1B in the formation of broad H3K4me3 domains and transcriptional regulation, thus providing valuable insights into the pathogenesis of these SETD1B-related diseases.

Based on recent observations in mouse embryonic stem cell models with acutely global loss of H3K4me3, presumably all forms of H3K4me3, two studies have revealed a functional link between H3K4me3 and Pol II occupancy.^33,34^ Additionally, a previous study had established a link between H3K4me3 and induced gene activation, in particular mechanistically through TAF3–H3K4me3 interaction and PIC assembly.^35^ Here, our study reveals the role of broad H3K4me3, a rarely studied H3K4me3 form, in promoting Pol II binding during mouse spermiogenesis in vivo. A potential mechanism for this interaction could involve the PHD finger of TFIID, known to recognize histone H3K4me3, which may preferentially bind broad H3K4me3 regions and stabilize PIC assembly.^35,36^ By serving as a platform for transcriptional machinery, broad H3K4me3 domains likely ensure the efficient transcription of genes critical for spermatogenesis and the correct timing of transcriptional transitions during development. We propose a model wherein SETD1B mediates spermatid broad H3K4me3 formation and regulates gene transcription through promoting more Pol II occupancy by competing with regular H3K4me3 (Fig. 6g). Our model is supported by the following observations: 1) genes marked by spermatid broad H3K4me3 domains exhibit significantly higher levels of expression, Pol II and TAF3 occupancy compared to genes marked by regular H3K4me3; 2) erasure of broad H3K4me3 upon loss of SETD1B results in a reduction in Pol II and TAF3 occupancies, and the down-regulated transcription of corresponding genes; 3) the erasure of broad H3K4me3 upon *Setd1b* loss leaves genome with only regular H3K4me3 promoters, and triggers a redistribution of Pol II from the original broad H3K4me3 sites to regular H3K4me3 domains. This Pol II redistribution leads to increased transcription of lower-expressing genes — an indirect effect of SETD1B loss. Such an indirect effect also contributed to the compromised expression timing of stage-specific genes during spermatid development, causing an “early gene up late” and “late gene up early” phenomenon (Fig. 7g), a function that has not been attributed to H3K4me3 before. The underappreciation of the H3K4me3 function in controlling transcription timing is likely due to a lack of study in a continuously developing model.

Comparing the previously reported diffuse and broad H3K4me3 domains in MII oocytes, which predominantly decorate intergenic and gene-scarce regions^39,41^ (Supplementary information, Fig. S13a–c), we demonstrate that RS broad H3K4me3 domains are primarily located at promoters and enhancers. This distinct genomic distribution suggests a more active transcriptional role in spermatids, promoting gene expression required for spermiogenesis. Despite this difference, our findings reveal a shared underlying mechanism: both oocyte and RS broad H3K4me3 domains compete with regular H3K4me3 regions for transcriptional machinery. This competition appears to be crucial for regulating proper gene expression output and timing during key developmental transitions.

In summary, our investigation underscores the significance of extensive dynamic alterations of genome-wide repressive histone modifications at key developmental stages, i.e., type B (key transition stage from mitosis to meiosis) and D (key transition stage from meiosis to spermiogenesis) stages, and distinct broad H3K4me3 domains throughout RS development. These alterations likely reflect the intricate epigenetic remodeling of male germ cells, which is crucial for accurate spatiotemporal control of transcription output during spermatogenesis. Furthermore, these modifications may contribute to the production of sperm in vitro with an appropriate epigenotype capable of supporting normal development. Specifically, our study highlights the crucial role of SETD1B in mediating broad H3K4me3 formation, facilitating Pol II recruitment, and controlling the accurate timing and fine-tuned output of stage-specific master gene expression in spermatids. Our study identifies RFX2 as a key transcription factor that is required for SETD1B to establish broad H3K4me3 domains critical for gene activation during spermiogenesis. We also acknowledge that RFX2 loss leads to a global reduction of both broad and regular H3K4me3 domains, resulting in stronger transcriptional and developmental defects compared to SETD1B loss alone. This suggests that RFX2 may influence additional H3K4me3 methyltransferases beyond SETD1B. These findings highlight the complex regulation of H3K4me3 during RS development, driven by the interplay between transcription factors and epigenetic regulators.

## Materials and Methods

### Experimental model and subject details

Mice used in this study were as follows: *Lin28*-YFP, *Vasa*-mCherry, *Stra8*-GFPCre, *Rfx2* KO and *Setd1b*-floxed mice. The *Lin28*-YFP, *Vasa*-mCherry and *Stra8*-GFPCre mice were described previously.^4,75^ The *Rfx2* KO mouse line was a gift from Dr. Chunsheng Han (Institute of Zoology, Chinese Academy of Sciences). The *Setd1b*-floxed mouse line was generated by Shanghai Biomodel Organism Co., Ltd. To generate the *Setd1b*-floxed line, wherein exon 5 of the *Setd1b* allele is flanked by *loxP* sites, two independent guide RNAs targeting *Setd1b* introns 4 and 5 were made. The donor vector, encompassing exon 5 flanked by two *loxP* sites and two homology arms, served as a template. Subsequently, *Setd1b*-floxed mice were crossbred with the germ cell-specific *Stra8*-GFPCre mouse line to yield germ cell-specific *Setd1b* KO mice. All mice described above were maintained on the C57BL/6 J (B6) background. All animal experiments were conducted following the guidelines of the Animal Care and Use Committee at the Center for Excellence in Molecular Cell Science, Chinese Academy of Sciences.

An adult human testicular sample for ChIP-seq was obtained from a healthy man (30 years old). The sample was removed from a deceased individual who had consented to organ donation for transplantation and research. The experiments performed in this study were approved by the Ethics Committee of the Center for Excellence in Molecular Cell Science, Chinese Academy of Sciences (2022-151).

### Mouse spermatogenic cell synchronization and purification

Spermatogenesis was synchronized and validated as previously described.^1^ Briefly, 2-dpp mice were pipette fed 100 μg/g body weight WIN 18,446 (MP Biomedicals, 158050), suspended in 1% gum tragacanth (Aladdin, G106434) daily for seven consecutive days to impede spermatogonial differentiation. On the day following the final WIN 18,446 treatment, these animals received an intraperitoneal injection of retinoic acid (25 μg/g body weight) (Sigma, R2625) in dimethyl sulfoxide (DMSO) (Sigma, D2650), to synchronously re-start spermatogonial differentiation, and the mice were then allowed to recuperate for sample collections. The synchronized germ cells can be collected any day from 0 to 30 days after retinoic acid injection based on the specific cell type of interest. The undifferentiated spermatogonia were isolated from *Lin28*-YFP knockin mice. Testes from 2-week-old *Lin28*-YFP knock in mice were collected and digested by type I collagenase (Worthington, LS004196) and 0.25% Trypsin (Gibco, 25200072) as described previously.^75^ After centrifugation, the pellet was resuspended in DMEM (Hyclone, SH30243.01) containing 2% BSA (Jackson ImmunoResearch, 001-000-161) at a concentration of 1 × 10^6^ cells/40 μL, followed by incubation with PE-conjugated anti-CD117 antibody (0.1 μg/10^6^ cells) (Invitrogen, 12-1171-82) for 30 min on ice. The undifferentiated spermatogonia (YFP-positive, PE-negative population) were collected using FACS (BD Aria II). The type A1 spermatogonia were isolated from synchronous *Stra8*-GFPCre mice. Testes were collected and digested. The type A1 spermatogonia (GFP-positive population) were collected at a given time-point 10 h after retinoic acid treatment using FACS. The type B spermatogonia and spermatocytes at different stages were isolated from synchronous *Lin28*-YFP and *Vasa*-mCherry double-positive mice. To isolate the type B spermatogonia, testes were digested and the synchronous advanced spermatogenic cells (mCherry-positive population) were collected at a given time-point 135 h after retinoic acid treatment using FACS. To isolate spermatocytes and spermatids at different stages, testes were digested and the cell suspensions were stained with Hoechst 33342 (Sigma, 14533). The mCherry-positive 1 N, 2 N–4 N or 4 N populations were collected using FACS. Specifically, spermatocytes at different stages were collected at the given time points of 168 h (preleptotene, pL), 186 h (leptotene, L), 208 h (zygotene, Z), 312 h (mid-pachytene, mP), and 384 h (diplotene, D) after retinoic acid treatment. Spermatids at different stages were collected at the given time points of 432 h (steps 1–2 RSs, RS2), 480 h (steps 3–4 RSs, RS4) 528 h (steps 7–8 RSs, RS8) and 576 h (steps 10–12 elongating spermatids, LS) after retinoic acid treatment. The developmental stages of spermatids were determined by the morphology of acrosome.^84^

### Human spermatogenic cell purification

Testes were collected and transported to the research laboratory on ice in PBS within 1 h. To facilitate the digestion process, the larger tissue samples were carefully divided into smaller sizes, approximately ranging from 500 mg to 1 g each, using scissors. Subsequently, single testicular cells were isolated through a two-step enzymatic digestion method, following a protocol with minor modifications.^74^ Initially, the testicular tissues were digested with type I collagenase for 5 min at 37 °C with gentle agitation (250 rpm). Following this, the mixture was vigorously shaken and incubated for an additional 3 min. To separate the tubules, the suspension was subjected to centrifugation at 200× *g* for 1 min and washed with PBS. Subsequently, the tubules were digested using 4.5 mL of 0.25% Trypsin and 20 kU DNase I (Sigma, DN25). The resulting suspension was triturated vigorously three to five times and incubated at 37 °C for 5 min. This process was repeated in 5-min intervals for a total of up to 15 min. The enzymatic digestion was halted by adding 10% FBS (Gemini, 900-108). Single testicular cells were obtained by passing the cell suspension through strainers with mesh sizes of 70 µm. The resulting cell suspensions were then stained with Hoechst 33342 and incubated at 37 °C for 30 min. Using FACS, the 1 N RS and 4 N (pachytene/diplotene spermatocytes, P/D) populations were collected for further analysis.

### Histological and immunohistochemical analyses

Testes/epididymides were fixed in Bouin’s buffer or 4% paraformaldehyde (PFA) (Sigma, P6148), embedded in paraffin and sectioned. Sections were deparaffinized, rehydrated, and stained with H&E. For IF analysis, sections were boiled in 10 mM sodium citrate buffer (pH 6.0) for 18 min, brought to room temperature, and washed in PBS with 0.1% Triton X-100 (PBST). The sections were then blocked with blocking buffer (10% donkey serum in PBST) for 60 min at room temperature and later incubated with the H3K4me3 (CST, 9751S; 1:200 dilution) or H3K4me1 (Active motif, 39297; 1:200 dilution) primary antibodies in blocking buffer overnight at 4 °C. On the following day, slides were washed three times for 10 min in PBST, Alexa Fluor 594-conjugated donkey secondary antibody (Jackson ImmunoResearch, 711-585-152; 1:500 dilution) and FITC-conjugated peanut agglutinin (PNA) (Sigma, L7381; 1:1000 dilution) diluted in blocking buffer were then added. After 60 min at room temperature, the sections were washed in PBST, mounted in Mounting Medium With DAPI (abcam, ab104139), and then analyzed by fluorescence microscopy (ZEISS, Axio Scope.A1).

### Nuclear spreading and immunofluorescence

Nuclear spreading and immunofluorescence were performed as described.^85^ For immunofluorescence analysis, the following primary antibodies were used: rabbit anti-SYCP3 (Abcam, ab15093), mouse anti-γH2AX (Millipore, 05-636). The spreading nuclei were then detected with Alexa Fluor 488-conjugated or 594-conjugated secondary antibodies mounted, and analyzed by fluorescence microscopy.

### Staining of sperm with mitotracker and PNA

Cauda epididymides from control and *Setd1b* cKO mutants were minced in 1 mL prewarmed DMEM. After 15 min at 37 °C, tissue was removed, and the sperm suspension was further incubated with 1 μL of Mitotracker Green FM (Invitrogen, M7514) for another 15 min. Sperm was smeared onto slides, allowed to air-dry, and fixed with 4% PFA in PBS (pH 7.4) for 30 min at room temperature. The slides were then washed in PBST for 10 min and incubated with FITC-conjugated PNA in a blocking buffer for 60 min at room temperature. After being washed with PBST for three times, the slides were mounted in Mounting Medium with DAPI, and then analyzed by fluorescence microscopy.

### TEM

Fresh testes/cauda epididymides were fixed in 2.5% glutaraldehyde in 0.1 M phosphate buffer (PB), pH 7.4, for 2 h at 4 °C, washed with PB, postfixed in 2% OsO_4_ for 1.5 h, dehydrated in a graded ethanol series before being transferred to acetone, and embedded in Poly/Bed 812. Ultrathin sections were taken with a Leica EM UC7 ultramicrotome, doubly stained with uranyl acetate and Reynold’s lead citrate, and then imaged on a FEI Tecnai G2 Spirit TEM (FEI Company) at 120-kV accelerating voltage.

### CASA Analysis

Cauda epididymides from control and *Setd1b* cKO mutants were minced in 200 μL prewarmed Tyrode’s Salts (Sigma, T2397) and then incubated at 37 °C for 15 min in an incubator with 5% CO_2_ in air to release the sperm. The supernatant was collected, and sperm counts and motility were evaluated using the CASA system (Hamilton Thorne, Beverly, USA). At least 10 fields were assessed for each specimen (*n* = 3 independent experiments), and the percentages of motile and progressively motile spermatozoa were determined.

### Western blot analysis

The sorted homogenous synchronous spermatogenic cells were lysed with 2× SDS loading buffer (100 mM Tris-HCl, pH 6.8, 4% sodium dodecyl sulfate, 0.2% bromophenol blue, 20% glycerol, 200 mM *β*-mercaptoethanol), followed by 95 °C heating for 5–10 min, and then equal amount of histone proteins were loaded on 4%–20% SDS-PAGE gels and transferred to nitrocellulose membrane (Pall corporation). The membranes were blocked with 5% non-fat milk for 30 min and then washed with TBST twice. Then the membranes were incubated with different antibodies separately in TBST with 5% BSA overnight at 4 °C. Then HRP-conjugated goat anti-rabbit IgG (or goat anti-mouse IgG) was added to the blots for 1 h at room temperature, and the membrane was developed with Smart-ECL super reagents (Smart lifesciences, S32500). The relative signal of each band was quantified by ChemiDoc Imaging Systems (Bio-rad). Antibodies used in western blot were listed in the ChIP-seq part.

### Reverse transcription and qPCR

Total RNA was purified as described for RNA-seq. 200 μg of total RNA was reverse-transcribed into cDNA using the PrimeScript RT reagent kit with gDNA eraser (Takara, RR047A) according to the manufacturer’s protocol. The cDNA was subjected to qPCR using the Hieff SYBR Green Master Mix (Yeasen, 11201ES03) according to the manufacturer’s protocol. For qPCR, a standard curve was used for quantitative assessment of mRNA levels and normalized to *Gapdh* mRNA. Primer sequences used for qPCR: *Gapdh* Fw: 5’-ACAGCAACTCCCACTCTTCCAC-3’, Re: 5’-AGTTGGGATAGGGCCTCTCTTG-3’. *Setd1b* Fw: 5’-TGTTGGTGAGCTGGATGCTA-3’, Re: 5’-GCCTTGTCCATAGGAGTTGG-3’.

### ChIP-Seq and ChIP-qPCR

ChIP assay was carried out as previously described.^86^ We used the same approach and the same amount of spike-in as in our previous work.^46^ The sorted homogenous synchronous spermatogenic cells were crosslinked with 1% formaldehyde for 10 min and then quenched by adding 125 mM glycine. Chromatin samples were lysed with lysis buffer (20 mM Tris-HCl, pH 8.0, 500 mM NaCl, 1 mM EDTA, 1% Triton X-100 and 0.1% SDS) and sonicated with Qsonica Q800. Histone-specific antibody was incubated with chromatin samples overnight at 4 °C. Antibodies used were listed below: H3K9me2 (Abcam, ab1220, lot: GR325223-4), H3K9me3 (Active motif, 39161, lot: 15617003), H3K4me1 (Active motif, 39297, lot: 19417002), H3K27ac (Active motif, 39133, lot: 20017009), H3K27me3 (Active motif, 39155, lot: 31917019), H3K27me3 (CST, 9733S, lot: 8), H3K36me3 (CST, 4909S, lot: 2), H3K4me3 (CST, 9751S, lot: 8), Pol II-NTD (CST, 14958S, lot: 4), TAF3 (Abcam, ab188332). About 5 × 10^4^ cells were used for H3K4me3 and H3K27ac ChIP, 1 × 10^5^ cells were used for H3K27me3 and H3K36me3 ChIP, and 3 × 10^5^ cells were used for H3K9me2, H3K9me3, Pol II-NTD and TAF3 ChIP. For the histone modification ChIP experiment, we used 0.5 µg spike-in antibody (Active motif, 61686) and 25 ng spike-in chromatin (Active motif, 53083) in this ChIP assay according to the manufacturer’s guidelines. For Pol II-NTD and TAF3 ChIP experiment, we used 100 ng human HEK293T chromatin as spike-in chromatin, and the ChIP was performed in buffer containing 20 mM Tris-HCl, pH 8.0, 300 mM NaCl, 1 mM EDTA, 1% Triton X-100 and 0.05% SDS. The protein–DNA complexes were immobilized on 15 µL protein A/G beads (Smart lifesciences, SA032005) and then washed three times with lysis buffer, twice with low salt buffer (10 mM Tris-HCl, 250 mM LiCl, 1 mM EDTA, 0.5% NP40, 0.5% Na-deoxycholate) and once with 10 mM Tris-HCl (pH 8.0). Decrosslinking was carried out in elution buffer (50 mM Tris-HCl, pH 8.0, 10 mM EDTA and 1% SDS) at 65 °C for at least 5 h. Proteinase K and RNase A digestions were performed at 55 °C for 1 h. DNA samples were purified with PCR extraction kit (QIAGEN, 28006). DNA samples were analyzed using real-time PCR and prepared for deep sequencing according to the manufacturer’s guidelines (Vazyme, ND607). Finally, libraries were pooled and sequenced on the Illumina Hiseq 2500 sequencer or NovaSeq 6000 sequencer with paired end sequencing. The ChIP-DNA was subjected to qPCR using the Hieff SYBR Green Master Mix (Yeasen, 11201ES03) according to the manufacturer’s protocol. Both mouse-specific primers and *Drosophila*-specific primers were used for each sample. Primer sequences used for ChIP-qPCR: H3K9me+ Fw: 5’-CAGCAAGCCTGGTGTTTGTA-3’, Re: 5’-TATCACCATGCCAAGCACAT-3’; mHoxa7-tss Fw: 5’-AGATGCGGAAACTGGCTTCG-3’, Re: 5’-CGGGCTTATACAATGTCAACAG-3’; mHoxa13-tss Fw: 5’-CCCTTCCATGTTCTTGTTGAG-3’, Re: 5’-CTATGACAGCCTCCGTGCTC-3’; MajSAT Fw: 5’-GGCGAGAAAACTGAAAATCACG-3’, Re: 5’-CTTGCCATATTCCACGTCCT-3’; MinSAT Fw: 5’-TTGGAAACGGGATTTGTAGA-3’, Re: 5’-CGGTTTCCAACATATGTGTTTT-3’; L1-LINE Fw: 5’-TGGCTTGTGCTGTAAGATCG-3’, Re: 5’-TCTGTTGGTGGTCTTTTTGTC3-’; IAP1 Fw: 5’CGCTCCGGTAGAATACTTAC3’, Re: 5’-TGCCATGCCGGCGAGCCTGT-3’; dCG5276 (spike-in primer) Fw: 5’-CGCCTTCGTACTCGTCCTAC-3’, Re: 5’-GACCACCATTGTCCAGACTC-3’.

### NOMe-seq

NOMe-seq assay was performed as previously described with some modifications.^46^ Briefly, ~1000 cells were transferred into a 0.2 μL PCR tube containing 7 μL of ice-cold lysate buffer (50 mM Tris-HCl, pH 7.4, 50 mM NaCl, 0.25 mM EDTA, 10 mM DTT, 0.25 mM PMSF and 0.5% NP-40, plus 2 pg λDNA). After gently vertexing, the cell lysate was kept on ice for 10 min. The GpC methyltransferase M. CviPI (NEB, M0227) and SAM were then added to the lysate to a final volume of 10 μL containing 1 U/μL M. CviPI and 160 μM SAM. The in vitro methylation of nuclei was performed by incubating the mixture in a thermocycler at 37 °C for 45 min followed by heating at 65 °C for 25 min to inactivate the enzyme activity. After in vitro methylation, 1 μL of 20 mg/mL protease (Qiagen, 19155) was added and the mixture was incubated for 3 h at 50 °C to release genomic DNA. The released genomic DNA was then bisulfite-converted using the EZ-96 DNA Methylation-Direct MagPrep (Zymo, D5045) according to the manufacturer’s instructions. Afterward, the purified DNAs were annealed using random nonamer primers with a 5’-biotin tag (5’-Biotin-CTACACGACGCTCTTCCGATCTNNNNNNNNN-3’) in the presence of Klenow fragments (3’–5’ exo-) (NEB, M0212). Then, the primers were digested by exonuclease I (NEB, M0293) and the DNA was purified using Agencourt Ampure XP beads (Beckman Coulter, A63880). Dynabeads M-280 Streptavidin (Invitrogen, 11205D) were then used to immobilize the newly synthesized biotin-tagged DNA strands, and the original bisulfite-converted DNA templates were removed. Second DNA strands were synthesized using Klenow fragment with random nonamer primers (5’-AGACGTGTGCTCTTCCGATCTNNNNNNNNN-3’). After washing, the beads were used to amplify libraries using 15 cycles of PCR with the universal primer and index primer (NEB, E7335). The amplified libraries were purified with Agencourt Ampure XP beads twice. Fragments from 300 to 800 bp were selected by agarose gel electrophoresis and purified by Zymoclean Gel DNA Recovery Kit (Zymo, D4008). Finally, libraries were pooled and sequenced on the Illumina HiSeq 2500 sequencer for 150-bp paired-end sequencing.

### RNA-seq

Total RNA was isolated from different stages of spermatogenic cells with Trizol reagent (Life Technologies, 15596-018). RNA integrity was assessed using Agilent 2100 Bioanalyser. 100 ng total RNA was used for library construction. rRNA removal and libraries of cDNA were constructed using the TrueSeq Stranded Total RNA Library Prep Kit (Illumina) following the manufacturer’s instructions. The libraries were sequenced on the Illumina HiSeq 2500 or Novaseq 6000 platform with 150 bp pair-end reads.

### Co-IP

The co-IP of murine RFX2 and SETD1B was conducted by exogenously overexpressing these proteins in HEK293T cells. Specifically, 0.5 μg of pCDNA3-SETD1B-HA plasmids and 1.5 μg of pLVX-Flag-RFX2 plasmids were co-transfected into HEK293T cells in a 6-well plate using Lipofectamine 2000. As a negative control, cells were transfected with 0.5 μg of pCDNA3-SETD1B-HA plasmids alone. After 48 h, cells were harvested into 1.5 mL tubes, washed twice with cold PBS, and then treated with benzonase nuclease (Smart-Lifesciences, SLP00800) in ice-cold HEPES buffer (20 mM HEPES-NaOH, pH 7.5, 20 mM NaCl, 0.1% Triton X-100) for 30 min to degrade RNA and DNA. Following nuclease digestion, approximately 200 μL of lysis buffer (20 mM HEPES-NaOH, pH 7.5, 150 mM NaCl, 0.1% Triton X-100, 1 mM PMSF, 1× protease inhibitor cocktail) was added to the tubes, and cells were lysed by sonication for 1 min. The cell lysates were centrifuged at 13,000 rpm for 10 min, and 10 μL of the lysate was saved as the input sample. The remaining supernatants were used for immunoprecipitation. For immunoprecipitation, pre-washed Flag-M2 beads were added to the lysates, and the tubes were rotated at 4 °C for 8 h. The beads were then washed three times with lysis buffer before proceeding to western blot analysis. Antibodies against the HA-tag and Flag-tag were used to detect SETD1B-HA and Flag-RFX2 proteins, respectively.

### ChIP-seq data processing

Low-quality bases and adapter-containing reads were first trimmed from all ChIP-seq raw data using a trim-galore package (version: 0.6.6) under default parameters,^87^ clean reads shorter than 30 bp were discarded. Trimmed ChIP-seq data were aligned to the reference genome of mouse mm10 or human hg19 assembly using bowtie2 (version: 2.4.1)^88^ with default parameters, mapped reads in sam files were converted to bam files using ‘samtools sort’,^89^ and duplicates were removed using ‘samtools rmdup’. All uniquely and mono-clonally mapped reads were extended to 150 bp using awk command. The spike-in reads were similarly aligned to the reference genome of *Drosophila* dm6 or human hg19 assembly using bowtie2. Uniquely mapped reads with 150-bp extension were further converted to genome coverage data (bedGraph format) with ‘bedtools genomecov’.^90^ bedGraph files were used to normalize based on spike-in reads, and normalized bedGraph files were then converted to bigWig files using public UCSC script (bedGraphToBigWig). We selected 30 M uniquely mapped reads at random to identify peaks, using the input sample as a control with MACS2 (version: 2.2.7.1) and epic2^91^ under default settings. Peaks for H3K4me3, H3K4me1, and H3K27ac were identified using MACS2 in regular mode (-qvalue 0.01). For H3K27me3, H3K36me3, and H3K9me3 peaks, we employed MACS2 in broad mode (--broad --broad-cutoff 0.01 --qvalue 0.01). Lastly, H3K9me2 peaks were determined using epic2 (--false-discovery-rate-cutoff 0.05). Considering that histone marks ChIP-seq data were highly reproducible between biological replicates, we merged uniquely mapped reads for two biological replicates for downstream analysis in this study. All of these metagene read density profiles and heatmaps in this study were plotted using deeptools plotProfile and plotHeatmap with the normalized histone marks genome coverage data (BigWig files). Of note, downstream analysis of ChIP-seq data was performed only for autosomes, but not for sex chromosomes. For all ChIP-seq datasets included in this study, we implemented a comprehensive quality control pipeline to ensure high data quality. First, all samples were required to meet a stringent mapping threshold, where at least 70% of reads were mapped to the reference genome (mm10). Additionally, we analyzed the distribution patterns of histone marks, confirming their expected enrichment patterns at promoters and gene bodies, ensuring that the ChIP-seq signals corresponded to biologically relevant genomic regions. We also ensured that the combined replicates for each histone mark had a sufficient number of uniquely mapped reads, with a threshold set at 30 million reads per replicate. This was essential for accurate peak calling and statistical power in downstream analyses. Finally, we performed manual inspections of the data using the UCSC Genome Browser to visually confirm the distribution and enrichment of ChIP-seq peaks at known regulatory regions.

### NOMe-seq data processing

Firstly, raw sequencing data were trimmed with 9-bp random primer, and adapters and low-quality bases were removed using software *trim_galore* (version: 0.1.3) with below parameters ‘-quality 20 -stringency 3 -length 50 -clip_R1 9 -clip_R2 9 -paired -trim1 -phred33’. Then, the clean reads were aligned against the mouse reference genome (UCSC, mm10) using the software *Bismark* (version: 0.7.6) with a paired-end and non-directional aligned mode.^92^ Additionally, the unmapped reads were re-aligned into the same reference genome with a single-end aligned mode to improve the number of mapped reads. Finally, PCR duplicated reads were removed from aligned BAM files using the software SAMtools (version: 0.1.18).

To improve the coverage and depth of NOMe-seq data, final BAM files from the same biological replications were merged for the downstream analysis. Only the detected cytosine site with a read depth greater than three was considered in this study. The methylation level or chromatin accessibility level of each cytosine site was defined as the number of methylated reads ‘C’ divided by the number of all detected reads (methylated and unmethylated reads, ‘C + T’). We used WCG (ACG/TCG) for DNAme analysis and GCH (GCA/GCC/GCT) for chromatin accessibility analysis.

### RNA-seq data processing

Raw RNA-seq reads were subjected to remove adapter contamination and low-qualified sequences using trim_galore (version: 0.6.6) under the paired-end mode, trimmed paired-end reads shorter than 30 were discarded. Trimmed RNA-seq data were aligned to the mouse genome build mm10 using hisat2 (version: 2.2.1)^93^ with default parameters (--add-chrname), mapped RNA-seq reads in sam files were converted to bam files using ‘samtools sort’. Mapped reads on transcripts of mm10 Refseq annotation were counted using htseq-count under the parameters: ‘-f bam -r pos -s no --nonunique all’ with bam files. Differentially expressed genes were identified using DEseq2 packages^94^ with the RNA-seq mapped reads counts on mm10 Refseq transcripts, under the parameters: Log_2_(fold change) >= 0.5 or <= –0.5, adjusted pvalue <= 0.05. FPKM values of transcripts were calculated using FPKM_count.py in RSeQC package.^95^ FPKM values of mm10 Refseq genes smaller than 0.5 were regarded as repressed genes. Volcano plots for differential expressed genes between adjacent stages were generated with EnhancedVolcano R package. *Z*-scores matrix for expressed genes (FPKM values) were calculated using normalize function in circlize R package. Clustered heatmap for all differential expressed genes identified during spermatogenesis were generated using ComplexHeatmap R package.

### Identification of NDRs

NDRs were individually identified with 3× coverage GCH sites for each sample. NDRs were defined as regions with significantly higher chromatin accessibility levels than those in the whole genome background. As previous reported,^96^ a 100-bp window with a 20-bp sliding step were used to call NDRs, and only regions matched the following three criteria were defined as NDRs: (1) the average chromatin accessibility level significantly higher than those in the whole genome background with *P* ≤  10^−10^ by χ^2^ test; (2) the number of GCH sites in the region greater than 5; and (3) the length of the region greater than 140 bp.

According to the distance from NDRs to transcription end site (TES), we defined NDRs as proximal NDRs and distal NDRs. NDRs located within 1.5 kb upstream and 1.5 kb downstream of the TSS were considered as proximal NDRs, and otherwise, as distal NDRs.

### Estimation of DNAme and chromatin accessibility levels

DNAme (WCG) and chromatin accessibility levels (GCH) were estimated in different genomic regions, and only regions with at least three WCG/GCH sites were considered in this study unless otherwise specified.

When we observed the similarity of DNAme of spermatogenic cells, we separated the whole genome into several 1-kb bins and calculated the average DNAme levels in each bin. When we observed the DNAme levels around the gene body which was defined as the region from the TSS to the TE, we separated each gene body into 100 bins. In addition, we extended the gene body region (5-kb upstream from TSS and 5 kb downstream from TES), and separated these extended regions into 10 bins with a size of 500 bp.

When we observed the chromatin accessibility level around the TSS, we extended the region 2 kb upstream and 2 kb downstream from the TSS, and separated each region into 200 bins with a size of 20 bp. The average chromatin accessibility level of each GCH site was individually obtained as the basic line for each stage. The chromatin accessibility level was quantified with the exclusion of the basic line in this study unless otherwise specified.

### Performing PCA based on DNAme and chromatin accessibility levels

When we performed PCA to observe the similarity of different spermatogenic cells based on DNAme levels, we separated the whole genome into 1-kb bins and then calculated the average DNAme levels in each bin with at least three WCG sites. The bin with undetected WCG level in any samples was excluded, and we performed PCA using function pca in R package pcaMethods with the probabilistic PCA method (parameter ‘ppca’).

The PCA based on chromatin accessibility level was performed based on the level in NDRs. After calling NDRs from each sample, we merged NDRs from all samples within 10 bp using subcommands multiinter and merge in software bedtools (version: 2.29.1). Then, for each sample, we calculated the chromatin accessibility level in each merged NDR with at least three GCH sites. The NDR with undetected GCH level in any samples was excluded, and PCA was performed using the same method described above.

### Performing PCA based on histone modifications

All ChIP-seq peaks of each histone mark at 11 consecutive developmental stages during spermatogenesis were merged to generate a combined peak list using ‘bedtools merge’ (-d -500). The normalized bigWig files were used to calculate the average read density at each merged peak with the “bigWigAverageOverBed” tool in deeptools, which was then used to generate the read density matrix. PCA was performed using the read density matrix with the R packages ‘factoextra’ and ‘FactoMineR’. For RNA-seq data, the FPKM value of each expressed gene was calculated using mapped reads (bam files) at 11 consecutive developmental stages, and the matrix of FPKM values for all expressed genes was used for PCA. For chromHMM chromatin states, each defined chromHMM state was assigned a score based on the chromatin state number, state 1 (Active promoter) was assigned the highest score (15), and state 15 (No signal) was assigned the lowest score (1). The mm10 reference genome was split into 5-kb tiling bins, averaged chromatin state score was calculated for each 5-kb tiling bin using the ‘bigWigAverageOverBed’ at 11 consecutive developmental stages during spermatogenesis. PCA was performed using the chromatin state score matrix.

### Metagene profiles and heatmaps for ChIP-seq

All of these metagene profiles and heatmaps in this study were plotted using deeptools plotProfile, and plotHeatmap with the normalized histone marks genome coverage data (BigWig files).

### Identifying the optimal number of *K*-means clusters

The optimal number of *K*-means clusters for the histone marks ChIP-seq data at each of the 11 consecutive stages of spermatogenesis was determined using the fviz nbclust function in the factoextra R package with the elbow method.^97^ This method allows for the identification of the optimal number of clusters by identifying an “elbow” point in a plot of within-cluster sum of squares (WCSS) values vs the number of clusters. The WCSS values are calculated for each number of clusters, and the optimal number of clusters is typically chosen as the point where the WCSS decreases more slowly.

### ChromHMM analysis

The method for identifying and characterizing chromatin states during spermatogenesis involved the use of ChromHMM (v1.23).^49,50^ These uniquely mapped files of H3K4me3, H3K4me1, H3K27ac, H3K27me3, H3K36me3, H3K9me3, and H3K9me2 ChIP-seq data at 11 developmental stages were split into 200-bp tiling bins using the BinarizeBam command, with the input alignment files as the control. Uniquely mapped reads from DSBs and PARs on X and Y chromosomes were excluded from the analysis. The ChromHMM model was trained with 2–24 emission states at a resolution of 200 bp with default parameters using the LearnModel command. The models with various numbers of emission states were compared to the model with 24 states. We then refer to the model learned with 7 histone marks using chromHMM reported in ENCODE project,^52^ and eventually derived a 15 emission chromatin states model with 7 histone modifications to annotate the genomes during mouse spermiogenesis. Finally, the genome was classified into the 15 emission chromatin states at each stage, with each state being assigned a descriptive label based on its similarity to known chromatin signatures and genomic distribution. The segmentation files for each stage were used to calculate the fraction of variable bases for each of the 15 emission chromatin states between two adjacent stages during mouse spermatogenesis.

### Bivalent gene definition

To identify bivalent domains during spermatogenesis, we identified overlapped H3K4me3 and H3K27me3 peaks at each stage. Bivalent genes were defined as those with bivalent domains on their promoters, which were defined as being within 2 kb of the TSS. To determine the overlap between H3K4me3 and H3K27me3 peaks, we used the ‘intersectBed’ tool in bedtools. As H3K27me3 peaks are typically broader than H3K4me3 peaks, a bivalent domain was defined as an H3K4me3 peak with at least one overlapping H3K27me3 peak.

### Broad H3K4me3 and H3K27ac domain definition

To define broad H3K4me3 and H3K27ac domains, for all H3K4me3 peaks at each stage during spermatogenesis in mice and humans, any two H3K4me3 peaks with a distance of no more than 500 bp were consolidated into a singular H3K4me3 peak using the command ‘bedtools merge -d -500’. Any merged H3K4me3 peaks exceeding 5 kb in length were classified as broad H3K4me3 domains. This same procedure was applied to identify broad H3K27ac domains in mice and humans. Notably, spermatid broad domains for H3K4me3 and H3K27ac, positioned 2 kb either upstream or downstream of the TSS in the mm10 Refseq annotation, were termed proximal broad H3K4me3 domains. All remaining domains were categorized as distal broad H3K4me3 domains.

### Classification of H3K4me3 peaks

To classify H3K4me3 peaks in RSs, we first merged H3K4me3 peaks using the method described above and filtered out any broad H3K4me3 peaks from the merged list. We then used the ‘bigWigAverageOverBed’ command to calculate the average H3K4me3 signals on the remaining merged peaks. The 500 merged H3K4me3 peaks with the highest peak heights were classified as sharp H3K4me3 peaks, while the remaining merged peaks were classified as control H3K4me3 peaks.

### Enhancer and SE definition

Active enhancers were identified by using distal H3K27ac peaks, at least 2 kb away from the TSS of mm10 Refseq genes. SEs were identified using rose (version: 0.176)^71^ with default parameters from H3K27ac ChIP-seq data at each stage during spermatogenesis. To quantify eRNA transcription from distal enhancers, we prepared RNA-seq libraries following standard protocols with ribosomal RNA depletion (ribominus treatment). This ensured the retention of non-polyadenylated RNAs, including eRNAs, during library preparation. For the analysis, sequencing reads were aligned to the mouse reference genome (mm10) using the Hisat2 aligner. eRNA transcripts were quantified as FPKM values using the FPKM_count.py script from the RSeQC package. Enhancers and SEs were defined based on H3K27ac ChIP-seq data, and eRNAs overlapping broad H3K4me3 and SE peaks were specifically compared to those from regular enhancers to generate the data presented in Fig. 4f.

### Assigning SEs and distal broad H3K4me3 peaks to their nearby genes

To identify the genes potentially regulated by SEs and distal broad H3K4me3 peaks, we used the gene annotation of mm10 NCBI RefSeq to assign these elements to their nearby genes. The distance from the center of enhancer to the TSS of each gene was calculated, and the gene closest to the enhancer and within a distance of 100 kb was considered potentially regulated by that SE or distal broad H3K4me3. This allowed for the comparison of the transcriptional activities of the genes potentially regulated by these elements.

### GO analysis

Functional annotation was performed using the Database for Annotation, Visualization and Integrated Discovery (DAVID) Bioinformatics Resource. GO terms for each functional process were summarized to a representative term, and –Log_10_(*P* values) were plotted with GraphPad Prism 7 to show the significance of each GO terms.

### GREAT analysis

GREAT analysis for broad H3K4me3 peaks was performed on the GREAT website (http://bejerano.stanford.edu/great/public/html/). Species assembly was set as “Mouse: GRCm38”, and the whole genome was chosen as background regions. Other parameters were set as default.

### Quantification and statistical analyses

All of the statistical analyses in this study were performed using R versions (http://www.r-project.org). For all figures with error bars, standard deviation (SD) was calculated and presented as mean ± SD. *P* value less than 0.05 was considered statistically significant (*P < 0.05; **P < 0.01; ***P < 0.001; ****P < 0.0001; ns, not significant). Comparisons between two or more groups were analyzed using an unpaired Student’s *t*-test. Fisher’s exact test was used to calculate the significance (*P* value) of whether two sets of peaks or genes are significantly correlated.

## Supplementary information

Supplementary Information, Table S1

Supplementary Information, Table S2

Supplementary Information, Table S3

Supplementary Information, Table S4

Supplementary Information, Table S5

Supplementary Information, Table S6

Supplementary Information, Table S7

Supplementary Information, Table S8

supplementary information

## Data Availability

All sequencing data including ChIP-seq, NOMe-seq, and RNA-seq data generated in this study are available at NCBI Gene Expression Omnibus (GEO) with the accession number: GSE242515. Histone marks ChIP-seq and NOMe-seq data derived from Undiff to D stage homogeneous populations of mouse spermatogenic cells are available at NCBI GEO with the accession number: GSE132446. H3K4me3 ChIP-seq data in mouse early embryo and human naïve CD4^+^ T cells are available at NCBI GEO with the accession numbers: GSE73952, GSM772836 and GSM772948, respectively. Other data that support the findings of this study are available from the corresponding author upon request.

## Code availability

Uniform processing pipelines for histone marks ChIP-seq data can be found here: https://github.com/Ruitulyu/KAS-Analyzer. Other source codes for NOMe-seq and RNA-seq data processing are available upon request.
