# Supplementary material for: SETD1B-mediated broad H3K4me3 controls proper temporal patterns of gene expression critical for spermatid development
Source: Cell Res. 2025 Mar 4;35(5):345–61. doi: 10.1038/s41422-025-01080-0 (PMC12012180; doi:10.1038/s41422-025-01080-0)
Supplement: Supplementary file 9 — supplementary information [file 41422_2025_1080_MOESM9_ESM.pdf]

Supplementary Information, Figure legends

Supplementary Information Figure S1

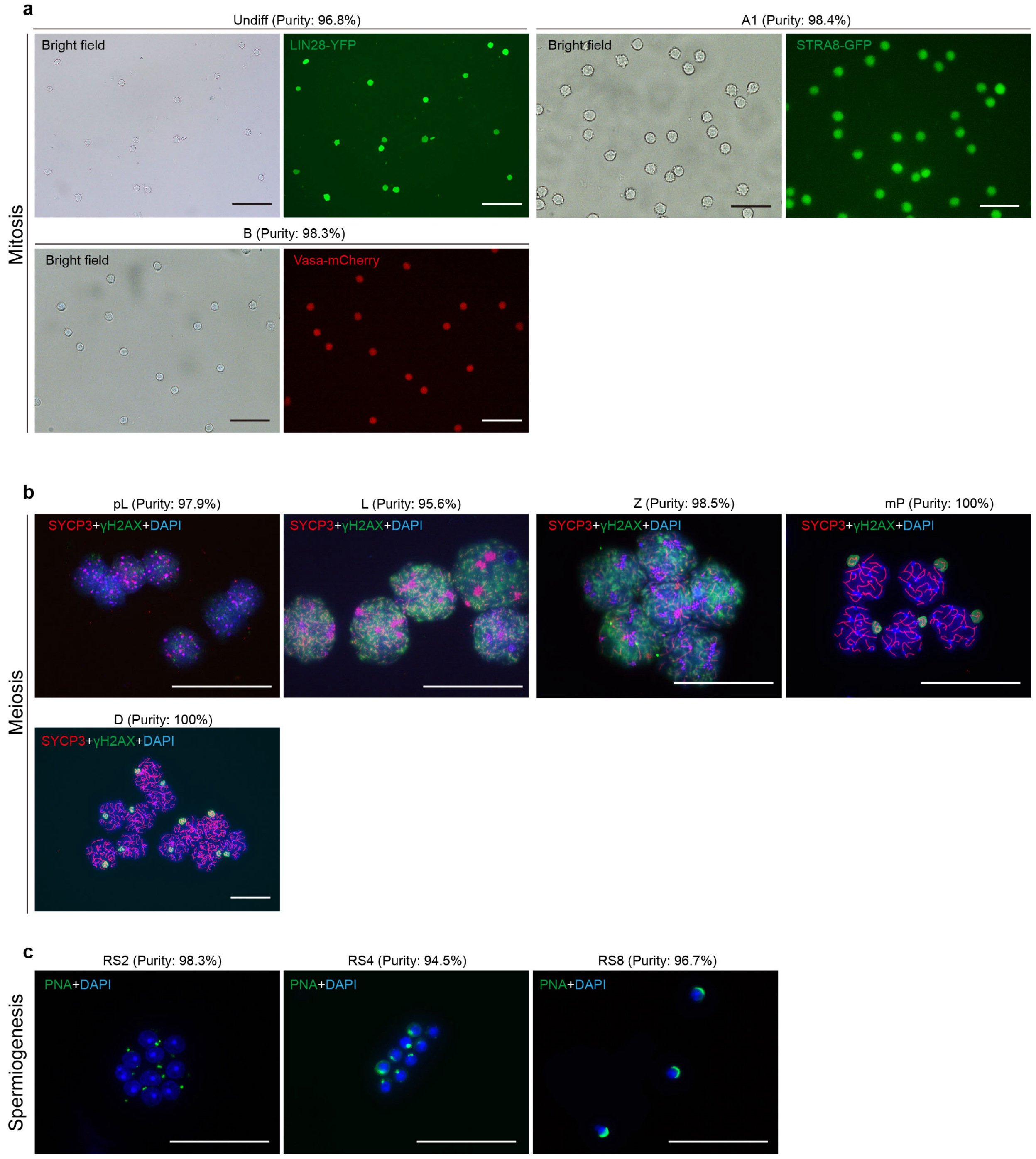

Supplementary Information, Fig. S1 Characterization of different stages of spermatogenic cells isolated for epigenomic analysis.

a, Brightfield and fluorescence images demonstrate the purity of different stages of mitotic cells (including Undiff, A1, and B). To ascertain the purity of Undiff, A1, and B, we evaluated their composition by observing the presence or absence of fluorescence

signals from the LIN28 marker (specifically expressed in undifferentiated spermatogonia), the STRA8 marker (specifically expressed in type A1 spermatogonia and preleptotene spermatocytes), and the MVH (specifically expressed in germ cells) marker, respectively. Scale bars: 100 um.

b, Surface-spread spermatocyte nuclei (immune)fluorescence staining for SYCP3 (the synaptonemal complex marker, red), γH2AX (the DNA damage signal, green) and DAPI of the sorted different stages of spermatocytes. The degree of homologous chromosome synapsis and DNA damage repair was used to ascertain the purity of different stages of spermatocytes. Scale bars: 100 um.

c, Fluorescence staining for PNA (spermatid acrosome marker) and DAPI of the sorted different steps of round spermatids. We assessed the morphology of the acrosomes to determine the cell stage and purity[97]. Scale bars: 100 um.

Supplementary Information Figure S2

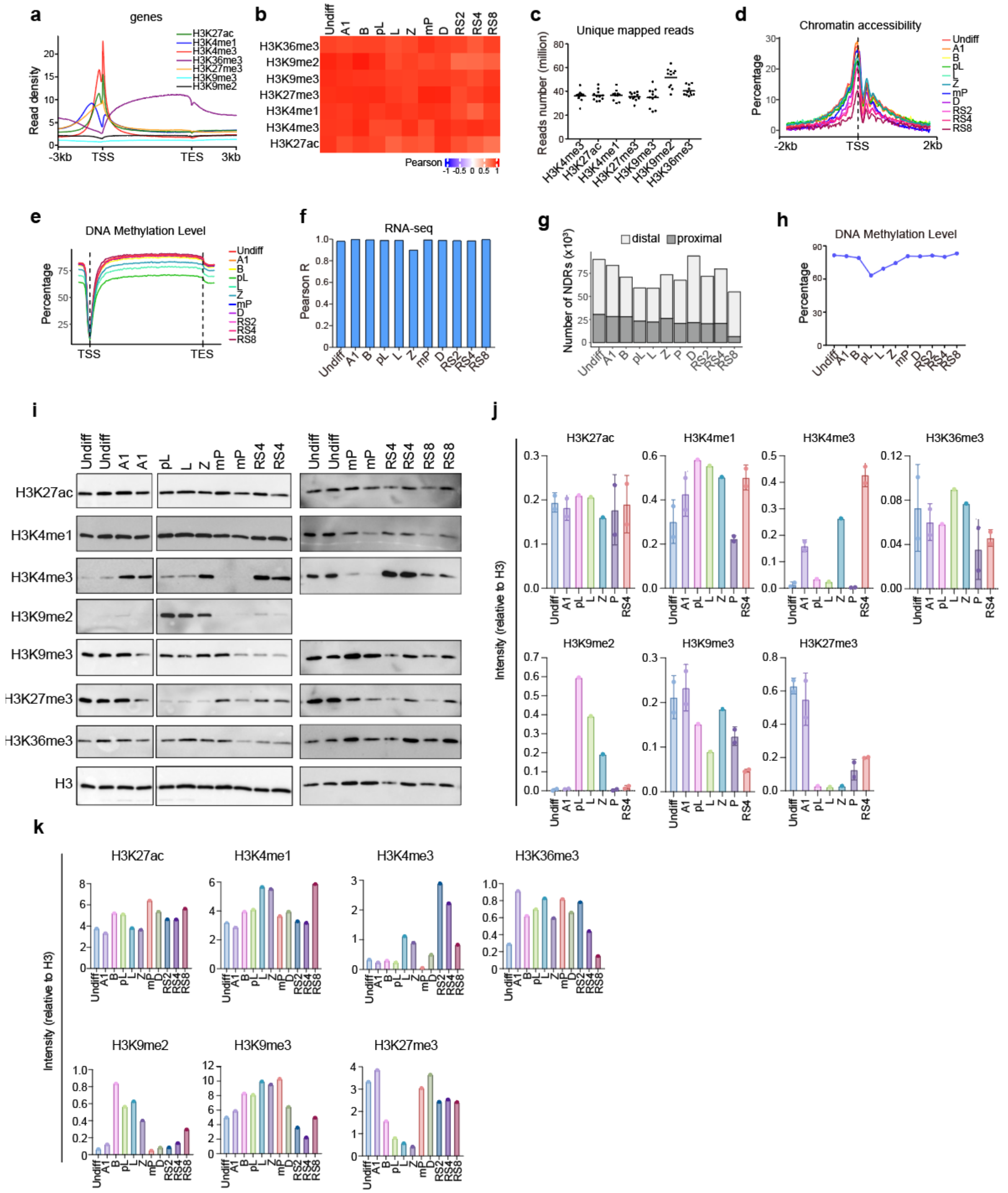

Supplementary Information, Fig. S2 Summary of ChIP-seq, NOMe-seq and RNA-seq data during mouse spermatogenesis.

a, Metagene profile plot showing the distribution of 7 different histone modifications at gene-coding regions during the RS4 stage, spanning from 3 kb upstream of the TSS to 3 kb downstream of the TES.

b, Heatmap showing the Pearson correlation coefficients between replicates for seven histone marks, as derived from ChIP-seq data generated across 11 stages of mouse spermatogenesis.

c, Scatter dot plot showing the number of uniquely mapped reads for 7 histone marks from ChIP-seq data across 11 stages of mouse spermatogenesis.

d, Metagene profile plot showing the averaged chromatin accessibility around TSS regions across 11 stages of mouse spermatogenesis.

e, Metagene profile plot showing the averaged DNA methylation levels at gene-coding regions across 11 stages of mouse spermatogenesis.

f, Bar plot showing the Pearson correlation coefficients between replicates for RNA-seq data across 11 stages of mouse spermatogenesis.

g, Stacked chart showing the number of nucleosome-depleted regions (NDRs) in proximal and distal regions across 11 stages of mouse spermatogenesis.

h, Line chart showing the global DNA methylation levels across 11 stages of mouse spermatogenesis.

i, Western blot analyses showing the global levels of histone marks during mouse spermatogenesis.

j, Quantification of histone marks based on western blot results in Fig. S2i.

k, Quantification of histone marks based on western blot results in Fig. 1d.

Supplementary Information Figure S3

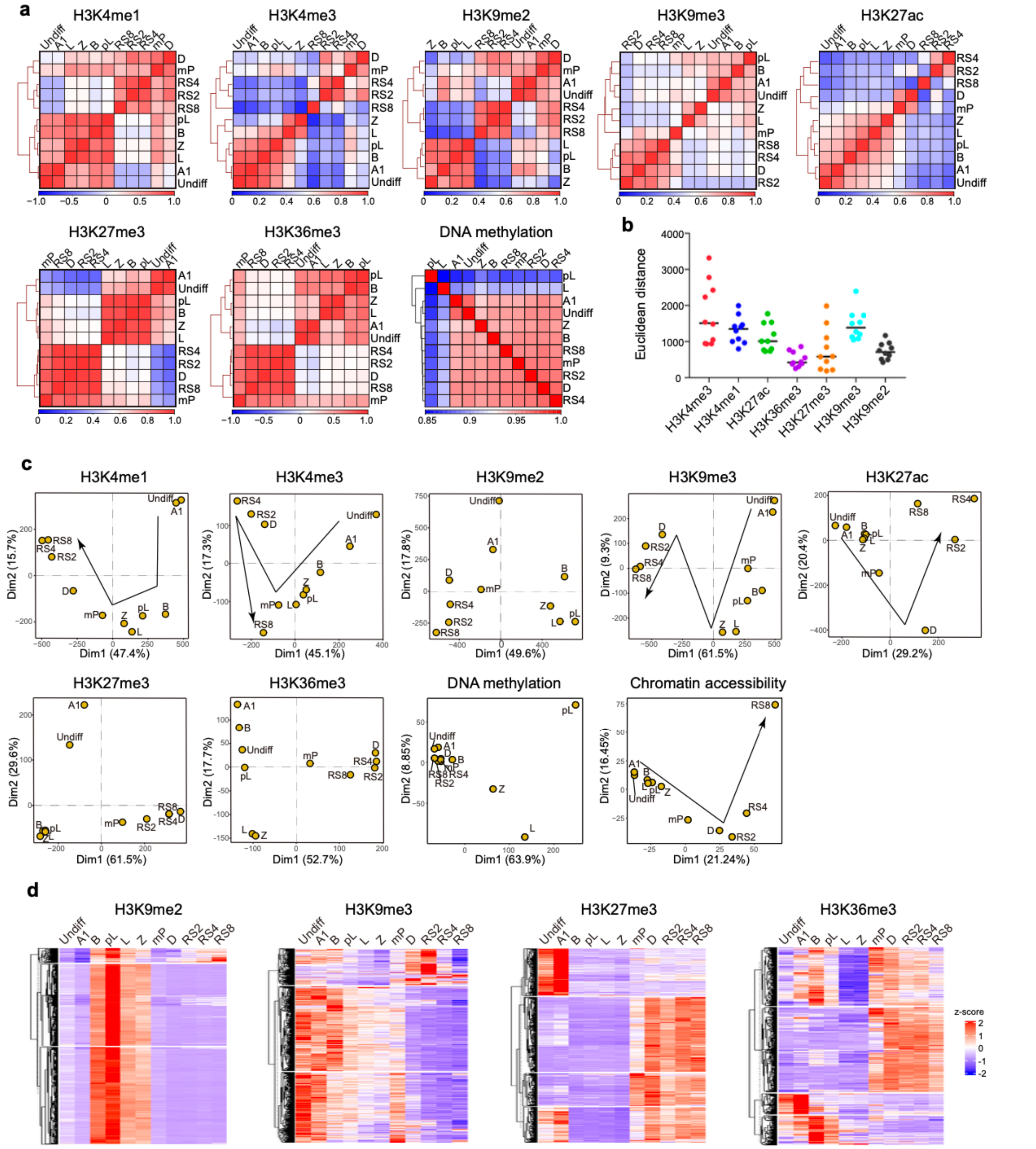

Supplementary Information, Fig. S3 Dynamic changes of chromatin during mouse spermatogenesis.

a, Clustered heatmaps showing the Pearson's correlation coefficients among 11 stages for 7 histone marks and DNA methylation.

b, Scatter dot plot showing the Euclidean distance of 7 histone marks during mouse spermatogenesis.

c, Principal component analysis (PCA) of 7 histone marks, DNA methylation, and chromatin accessibility across 11 stages of mouse spermatogenesis, with an arrowed line indicating the developmental progression of cells through various stages of spermatogenesis.

d, Clustered heatmaps showing the normalized read densities of H3K9me2, H3K9me3, H3K27me3 and H3K36me3 peaks across 11 stages of mouse spermatogenesis.

Supplementary Information Figure S4

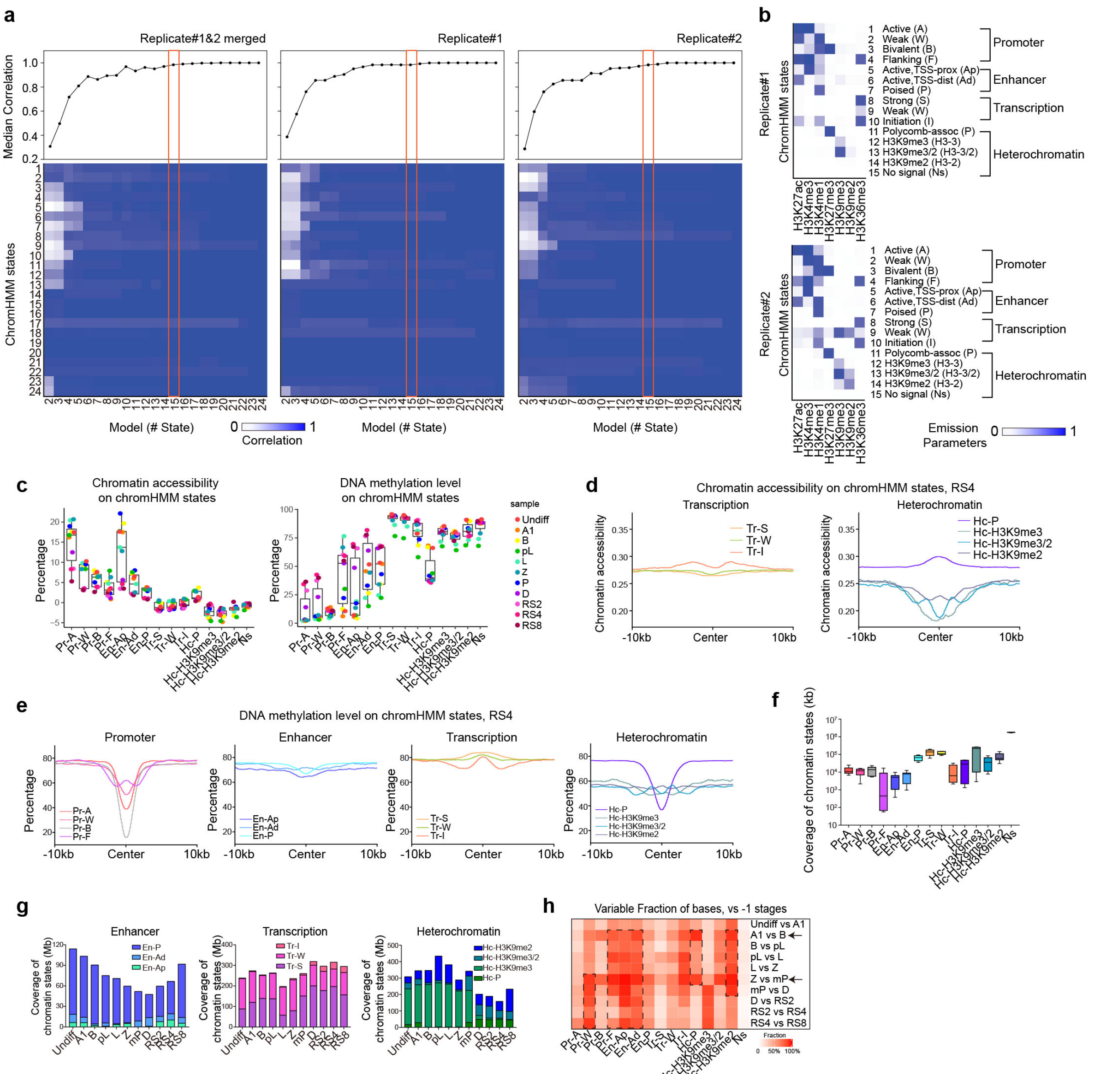

Supplementary Information, Fig. S4 ChromHMM model analyses based on 7 histone marks.

a, Heatmaps depicting the maximum Pearson's correlation of each state in the full model (y-axis) with its best matching state in each simpler model (x-axis) for merged data and two biological replicates. The median correlation of all 24 states is shown in the plots on top of the heatmaps. The colors in the heatmaps indicate the correlation strength, where dark blue represents a strong correlation, and light blue represents a weak correlation.

b, The emission probabilities for each chromatin mark in each state, as defined by chromHMM, for both biological replicates.

c, Box plots showing the levels of chromatin accessibility (left panel) and DNA methylation (right panel) on 15 chromHMM states across all 11 stages of mouse spermatogenesis. The boxes represent the interquartile range, with the median indicated by the horizontal line.

d, Metagene profile plots showing the averaged chromatin accessibility on 2 groups of chromatin states (transcription and heterochromatin) at RS4 stage.

e, Metagene profile plots showing averaged DNA methylation levels on 4 groups of chromHMM chromatin states at RS4 stage.

f, Boxplot showing the genomic coverage of 15 chromatin states across 11 stages during mouse spermatogenesis.

g, Stacked chart showing the genomic coverage of chromatin states related to enhancers (En), transcription (Tr), and heterochromatin (Hc) across 11 stages of mouse spermatogenesis. The height of each segment represents the genomic coverage by the specific state at each stage, with different colors indicating different chromHMM chromatin states.

h, Heatmap showing the fraction of bases for each state that vary between adjacent stages during mouse spermatogenesis.

Supplementary Information Figure S5

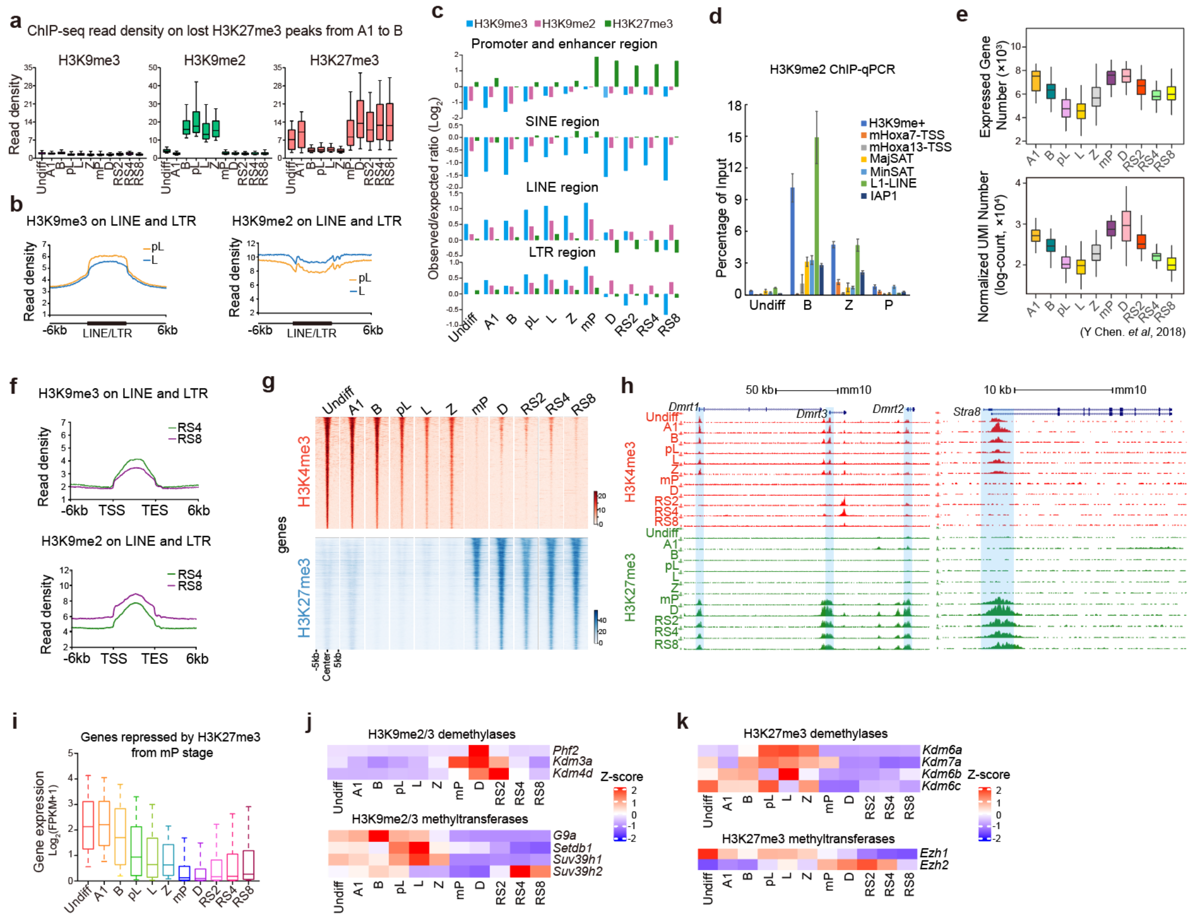

Supplementary Information, Fig. S5 Characterization of repressive modifications during spermatogenesis.

a, Box plots showing the normalized ChIP-seq read densities of H3K9me3 (left panel), H3K9me2 (middle panel), and H3K27me3 (right panel) on lost H3K27me3 peaks from A1 to B stages of mouse spermatogenesis. The central lines of the boxes represent the median values, the edges of the boxes indicate the interquartile ranges.

b, Metagene profile plots showing the normalized ChIP-seq read densities of H3K9me3 (right panel) and H3K9me2 (left panel) on LINE and LTR elements at pL and L stages during mouse spermatogenesis. The x-axis represents the genomic regions relative to the LINE and LTR elements.

c, Grouped bar plots showing the enrichment of H3K9me3, H3K9me2, and H3K27me3 in the indicated genomic regions, including promoter, enhancer, SINE, LINE, and LTR elements. The observed/expected Log2 ratios are used to quantify the degree of enrichment.

d, H3K9me2 ChIP-qPCR at gene promoter and repeats elements. (The ChIP-qPCR primer H3K9me+ referred to the gene desert downstream of Hoxa gene cluster, which is marked by H3K9me3 modification).

e, Hierarchical clustering heatmap showing the stage-specific gene expression pattern during mouse spermatogenesis.

f, Metagene profile plots showing the normalized ChIP-seq read densities of H3K9me3 (right panel) and H3K9me2 (left panel) on LINE and LTR elements at both RS4 and RS8 stages during mouse spermatogenesis. The x-axis represents the genomic regions around the LINE and LTR elements.

g, Heatmap showing the dynamic changes of the normalized ChIP-seq read densities of H3K4me3 and H3K27me3 at regions exhibiting H3K4me3 to H3K27me3 conversion at mP stage.

h, Snapshot of UCSC genome browser showing the normalized ChIP-seq read densities of H3K4me3 (red) and H3K27me3 (green) on the promoters of four representative genes. The *Dmrt* gene family (left panel) and *Stra8* (right panel) are presented as examples of genes expressed at the early stages of spermatogenesis (Undiff-Z). Regions showing H4K4me3 to H3K27me3 conversion at mP stage are highlighted by light blue shading.

i, Box plots showing the expression levels of genes displaying H3K4me3 to H3K27me3 conversion at mP stage across 11 stages of mouse spermatogenesis. The central lines of the boxes represent the median values, the edges of the boxes indicate the interquartile ranges. The gene expression levels were determined by RNA-seq analysis.

(j-k) Heatmaps showing the dynamic expression patterns of H3K9me2/3 (**j**) and H3K27me3 (**k**) demethylases and methyltransferases across 11 stages of mouse spermatogenesis. The heatmaps are plotted utilizing the Z-scores of the gene expression levels of those genes.

Supplementary Information Figure S6

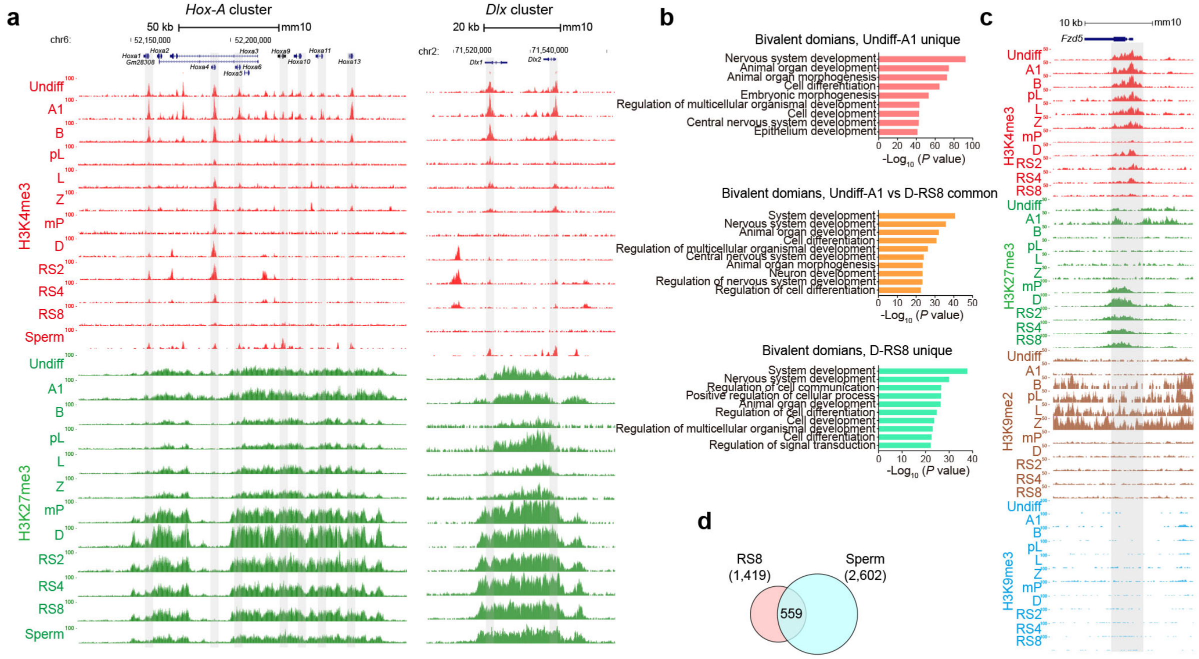

Supplementary Information, Fig. S6 Dynamics of bivalent domains during mouse spermatogenesis.

a, Snapshots of UCSC genome browser showing normalized ChIP-seq read densities of H3K4me3 (red) and H3K27me3 (green) on the *Hox-A* gene cluster and *Dlx* gene cluster during mouse spermatogenesis. The light gray shading highlights the promoters of bivalent genes, which are marked by both H3K4me3 and H3K27me3.

b, Gene ontology (GO) analysis of bivalent domains target genes during mouse spermatogenesis. The upper panel shows GO analysis for genes specific to Undiff and A1 stages. The middle panel shows GO analysis for genes common to both Undiff-A1 and D-RS8 stages. The bottom panel shows GO analysis for genes exclusive to the D-RS8 stage.

c, Snapshot of UCSC genome browser showing the normalized ChIP-seq read densities of H3K4me3 (red), H3K27me3 (green), H3K9me2 (brown) and H3K9me3 (blue) on *Fzd5* gene locus during mouse spermatogenesis. The light gray shading highlights the bivalent promoters, which are marked by both H3K4me3 and H3K27me3 during most stages of spermatogenesis except for stages B to Z, during which H3K9me2 replaced H3K27me3 resulting in the formation of atypical bivalency.

d, Venn diagrams showing the intersection of bivalent genes observed at the RS8 stage and in mature sperms.

Supplementary Information Figure S7

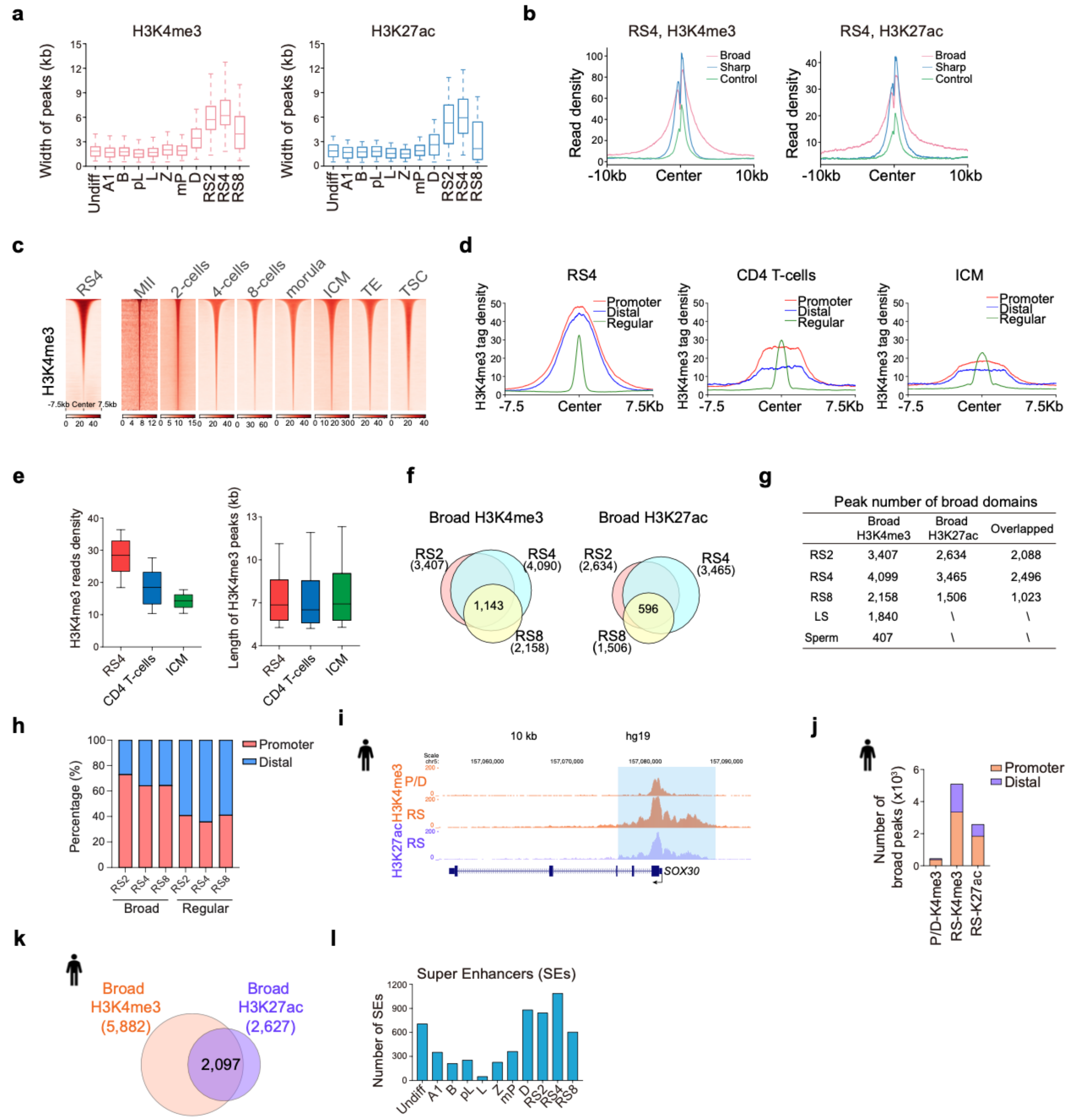

Supplementary Information, Fig. S7 Characterization of spermatid broad H3K4me3 domains.

a, Box plots showing the width of broad H3K4me3 (left panel) and H3K27ac (right panel) domains identified in round spermatids (RS) during mouse spermatogenesis. Broad domains are defined as peaks longer than 5 kb.

b, Metagene profile plots showing the normalized ChIP-seq read densities of H3K4me3 (left panel) and H3K27ac (right panel) on broad, sharp, and control peaks at RS4 stage.

c, Heatmaps showing the normalized ChIP-seq read densities on all H3K4me3 peaks sorted by peak width in RS4 and other indicated cell types. Peaks are sorted using width from broadest to narrowest.

d, Metagene profile plots showing the normalized ChIP-seq read densities of H3K4me3 on promoter or distal broad H3K4me3 peaks, as well as regular H3K4me3 peaks, in RS4 round spermatids, the inner cell mass (ICM) of early-stage mouse embryos, and human CD4+ T cells.

e, Box plots showing the normalized ChIP-seq read densities (left) and peak width (right) on broad H3K4me3 peaks in RS4 round spermatids, the inner cell mass (ICM) and human CD4+ T cells.

f, Venn diagrams showing the numbers of common broad H3K4me3 (left panel) and H3K27ac (right panel) peaks between RS2, RS4, and RS8 stages of round spermatids.

g, Table displaying the number of broad H3K4me3, broad H3K27ac, and broad H3K4me3 and H3K27ac overlapping peaks across RS2, RS4, RS8, LS, and mature sperm.

h, Stacked chart showing the distribution of broad H3K4me3 and regular H3K4me3 peaks in promoter regions and distal enhancer regions.

i, A snapshot of UCSC genome browser showing the normalized ChIP-seq read densities of H3K4me3 and H3K27me3 on the *SOX30* gene locus from human pachytene/diplotene spermatocytes (P/D) and round spermatids (RS). The light blue shading highlights the broad H3K4me3 peaks identified in human round spermatids.

j, Stacked chart showing the numbers of human broad H3K4me3 (P/D and RS) and H3K27ac peaks (only RS).

k, Venn diagrams showing the number of overlapped broad H3K4me3 and H3K27ac domains in human round spermatids.

l, Bar plot showing the numbers of super enhancers (SEs) identified by H3K27ac ChIP-seq data across 11 stages of mouse spermatogenesis using ROSE algorithm.

Supplementary Information Figure S8

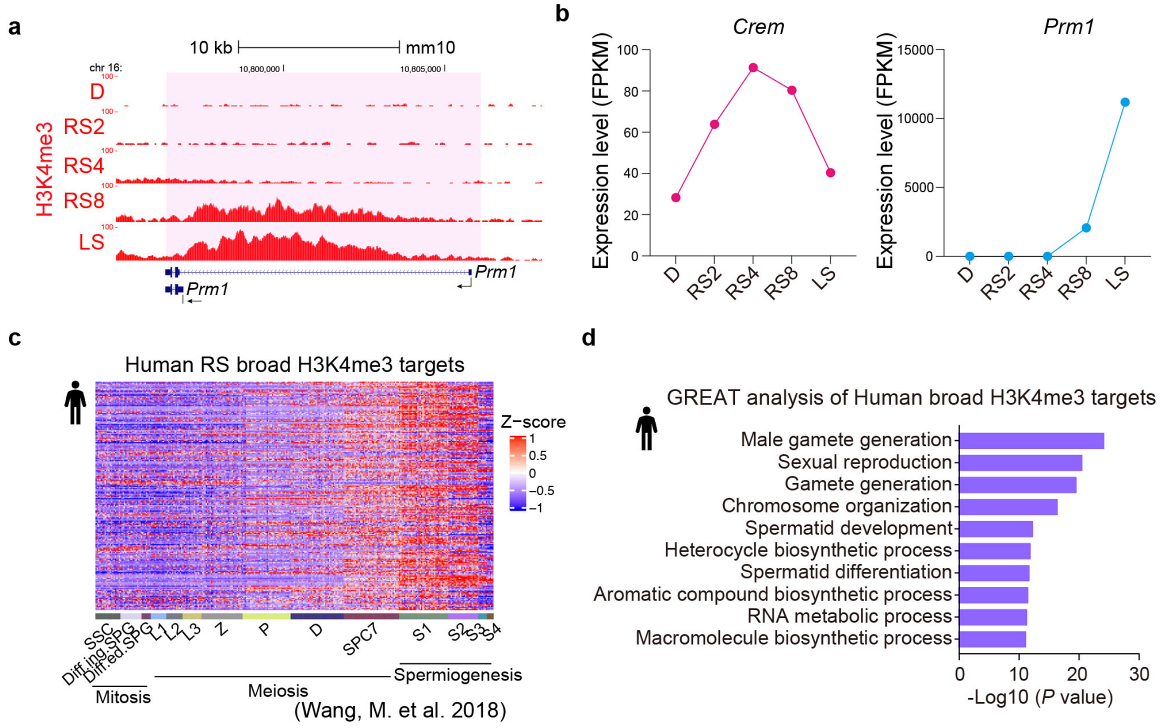

Supplementary Information, Fig. S8 The conserved role of spermatid broad H3K4me3 domains in transcriptional activation during spermiogenesis

a, A snapshot of UCSC genome browser showing the normalized ChIP-seq read densities of H3K4me3 over the *Prm1* gene locus during mouse spermatogenesis from D to LS stages. The light red shading highlights the broad H3K4me3 peaks identified in mouse round spermatids.

b, Point line chart showing the expression levels (in FPKM) of the representative broad H3K4me3 target genes, *Crem* and *Prm1*, throughout various stages of mouse spermatogenesis, from the D stage through to the LS stage.

c, Heatmaps showing the RNA expression levels of broad H3K4me3 target genes in human spermatids. The single-cell RNA-seq data was collected from Wang, M. *et al*. 2018.

d, Functional enrichment analysis of broad H3K4me3 in human spermatids utilizing GREAT to infer potential functional implications and associated biological processes.

Supplementary Information Figure S9

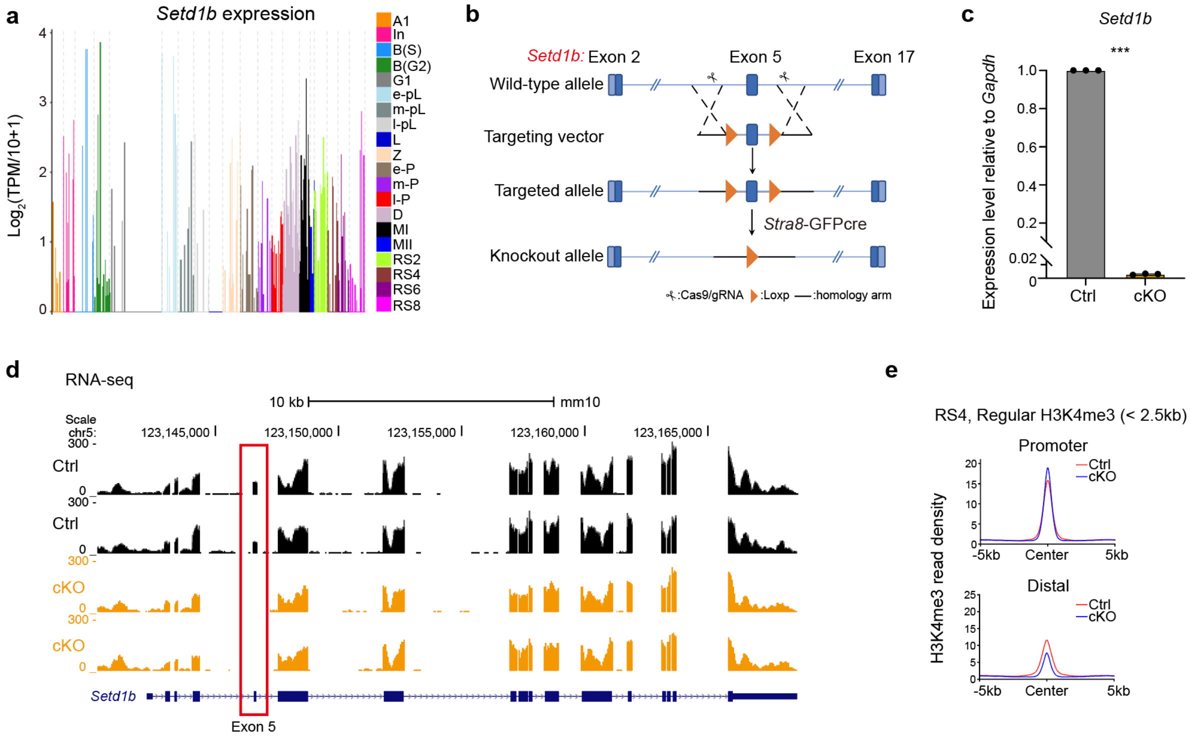

Supplementary Information, Fig. S9 Spermatid broad H3K4me3 domains are mediated by SETD1B.

a, Single-cell RNA-seq data showing the gene expression levels (TPM) of *Setd1b* during mouse spermatogenesis.

b, Strategies for generating *Setd1b* germ cell-specific knockout mice. Exon 5 of *Setd1b* was flanked by two *LoxP* sites.

c, Snapshots of the UCSC genome browser showing the normalized RNA-seq read densities on *Setd1b* gene locus. Exon 5 of *Setd1b* was highlighted in a red box.

d, qPCR results showing the relative expression level of *Setd1b* exon 5 in control and *Setd1b* *c*KO RS.

e, Metagene profile plots showing the normalized ChIP-seq read densities of H3K4me3 on promoter (upper) and distal (lower) regular H3K4me3 peaks (width < 2.5 kb) in control and *Setd1b* *c*KO RS4.

Supplementary Information Figure S10

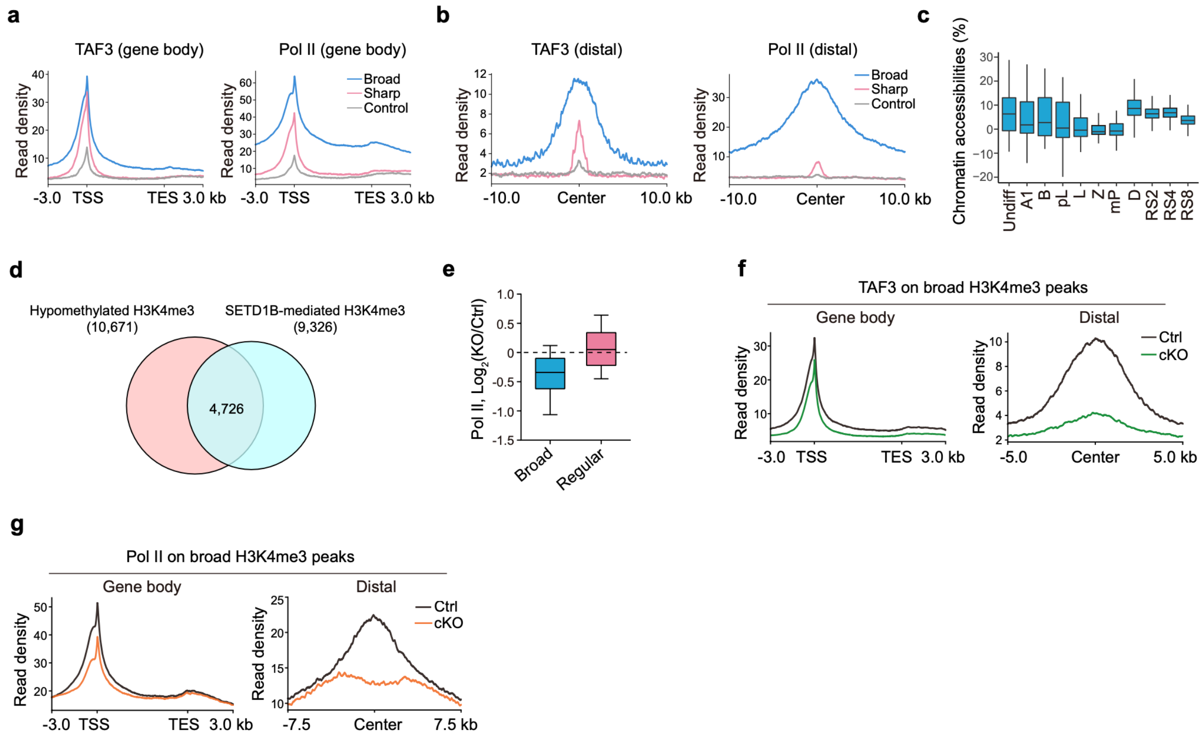

Supplementary Information, Fig. S10 SETD1B-mediated broad H3K4me3 promotes Pol II occupancy.

a, Metagene profile plot showing the averaged ChIP-seq read densities of TAF3 (left panel) and RNA Pol II (left panel) on broad, sharp, and control H3K4me3 marked genes in mouse round spermatids.

b, Metagene profile plots showing the normalized ChIP-seq read densities of TAF3 (left panel) and Pol II (right panel) on distal broad, sharp, and control peaks in round spermatids.

c, Box plot showing the chromatin accessibilities of broad H3K4me3 domains across 11 stages of mouse spermatogenesis.

d, Venn diagrams showing the overlapping between hypomethylated H3K4me3 domains and SETD1B-mediated H3K4me3 domains;

e, Box plots showing changes in RNA Pol II enrichment on all broad H3K4me3 target genes and regular H3K4me3 target genes in bulk RS.

f, Metagene profile plots showing the normalized ChIP-seq read densities of TAF3 binding on gene body (left panel) and distal (right panel) broad H3K4me3 domains in control and *Setd1b* *c*KO bulk RS.

g, Metagene profile plots showing the normalized ChIP-seq read densities of RNA Pol II binding on gene body (left panel) and distal (right panel) broad H3K4me3 domains in control and *Setd1b* *c*KO bulk RS.

Supplementary Information Figure S11

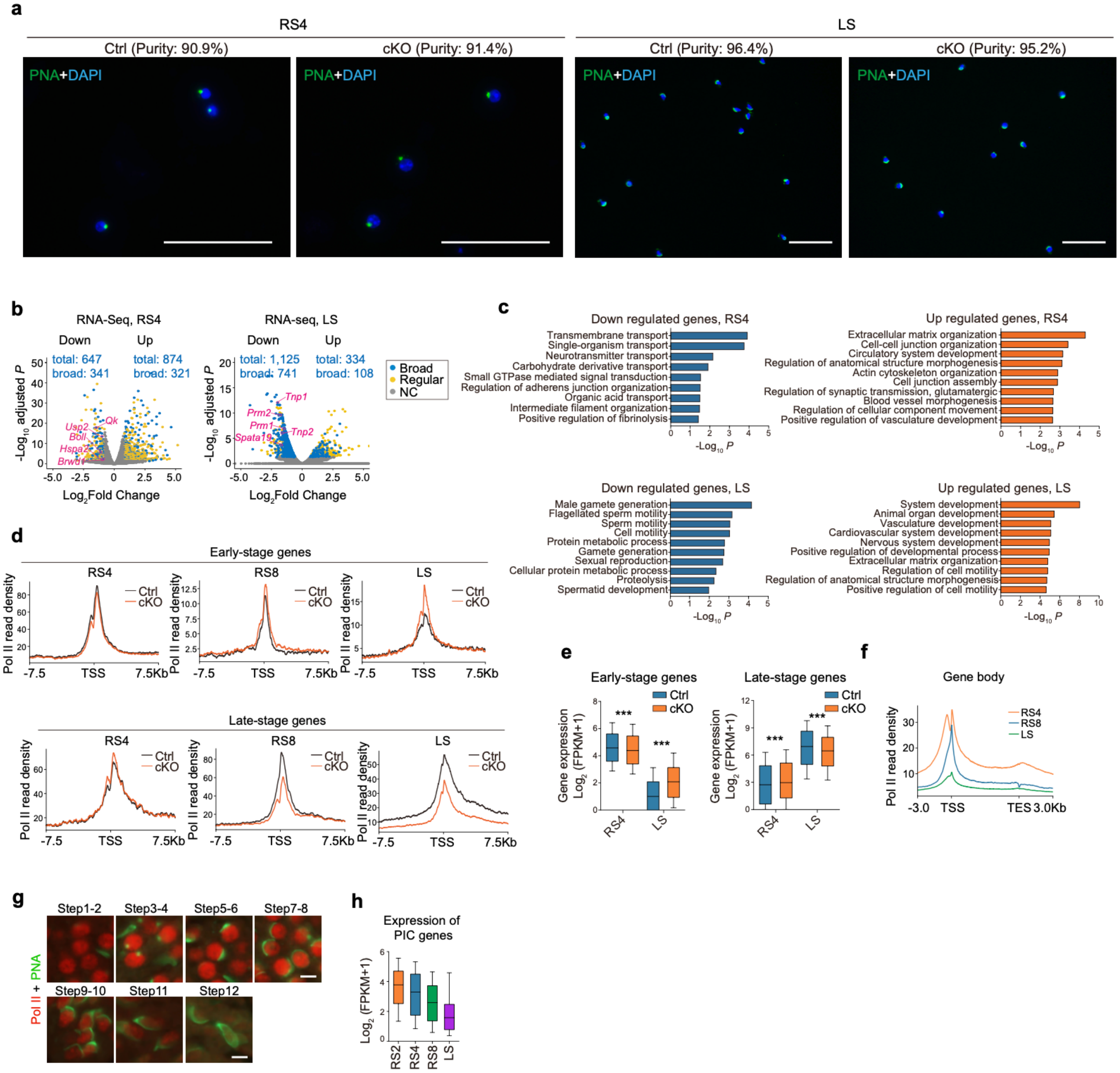

Supplementary Information, Fig. S11 SETD1B-mediated broad H3K4me3 ensures accurate expression timing of stage-specific genes and spermatid development.

a, Fluorescence staining for PNA (spermatid acrosome marker) and DAPI of the sorted different steps of RS4 round spermatids (left) and LS elongating spermatids (right) from control and *Setd1b* cKO mice. We assessed the morphology of the acrosomes to determine the cell stage and purity. Scale bars: 100 um.

b, Volcano plot showing the number of differentially expressed genes in RS4 (left panel) and LS (right panel) stages between control and *Setd1b* cKO mice. Blue dots represent differentially expressed broad H3K4me3 peaks target genes, and yellow dots represent differentially expressed regular H3K4me3 peaks target genes. Significance was defined by a threshold of Log_2 Fold change >= 0.5 or <= -0.5 and adjusted *P* value < 0.05. Representative down-regulated broad H3K4me3 genes are highlighted in the plot.

c, Gene ontology (GO) analysis of down- and up-regulated genes in the RS4 (upper two panel) and LS (lower two panel) stages between control and *Setd1b* cKO mice.

d, Metagene profile plots showing the normalized RNA Pol II read densities on early-stage and late-stage broad H3K4me3 target genes across RS4, RS8 and LS stages in control and *Setd1b c*KO mice.

e, Box plots showing the gene expression levels of early-stage and late-stage broad H3K4me3 target genes across RS4, RS8 and LS in control and *Setd1b c*KO mice. Early stage genes: RS4 ($^{***}P$ = 3.22479E-35), LS ($^{***}P$ = 1.0988E-149); Late stage genes: RS4 ($^{***}P$ = 5.37E-23), LS ($^{***}P$ = 7.81E-58). Paired two-tailed *t*-test.

f, Metagene profile plots showing the normalized ChIP-seq read densities of RNA Pol II binding on the gene body of broad H3K4me3 target genes at RS4, RS8, and LS stages during mouse spermatogenesis.

g, Immunofluorescent (IF) staining for Pol II (red) in sections of adult wild-type testes. Acrosome marker peanut lectin (PNA, green) was co-stained to determine the specific steps of spermatids. Scale bar, 10 μm.

h, Box plot showing the expression levels of transcription preinitiation complex (PIC) genes in the RS4, RS8, and LS stages of mouse spermatogenesis.

Supplementary Information Figure S12

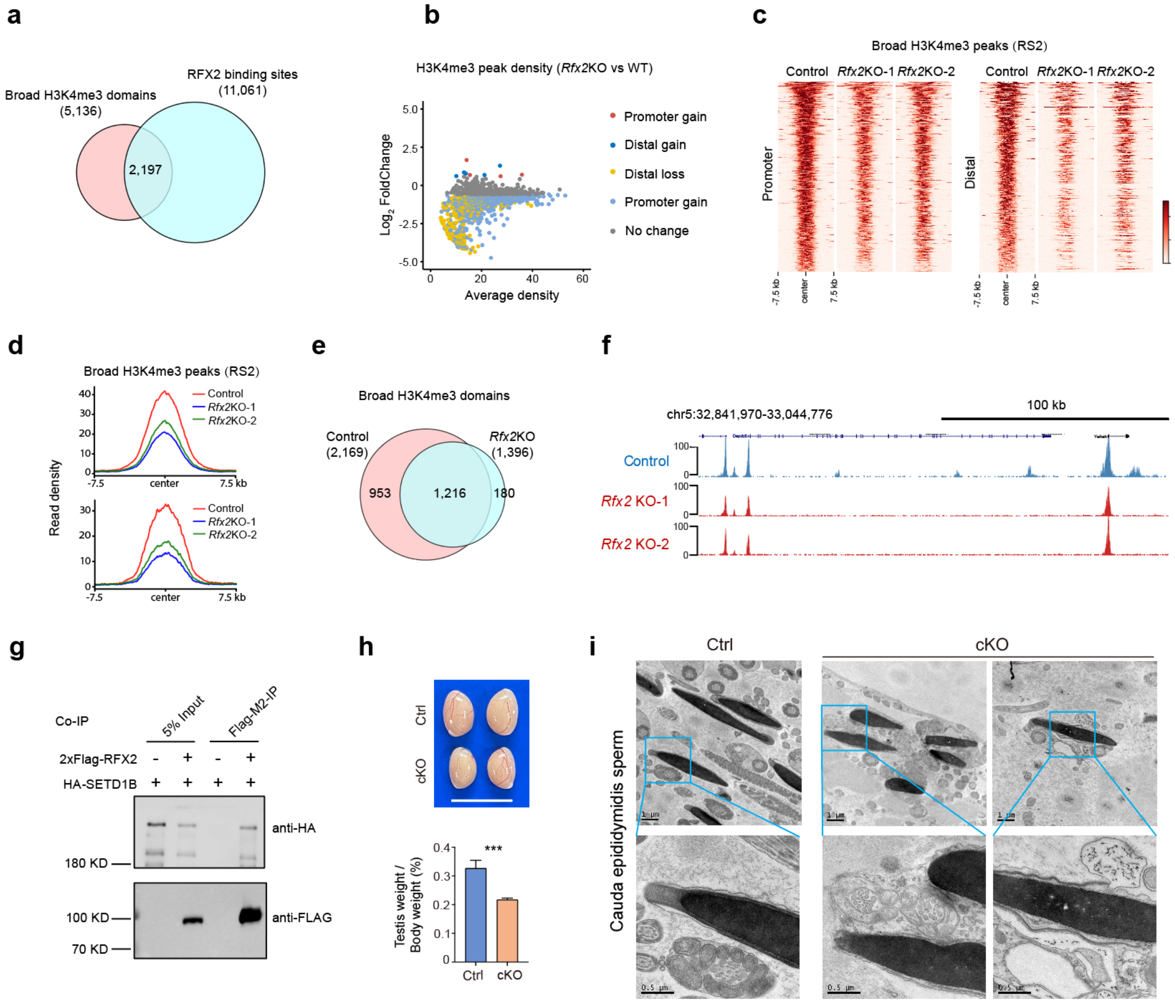

Supplementary Information, Fig. S12 Role of RFX2 in Recruiting SETD1B for Broad H3K4me3 Specificity During Spermiogenesis

a, Venn diagrams showing the overlapping between broad H3K4me3 domains and RFX2 inding sites;

b, MA plots showing changes in H3K4me3 density at promoter and distal regions in Control and *Rfx2* KO RS2. |Foldchange| >=1.5;

c, Heatmaps showing the normalized read densities of H3K4me3 across all broad H3K4me3 domains identified at the RS2 stage of round spermatids in both control and *Rfx2* KO mice. Broad domains are categorized into promoter (left panel) and distal (right panel) groups based on their genomic locations;

d, Metagene profile plots showing the normalized read densities of H3K4me3 across all broad H3K4me3 domains identified at the RS2 stage of round spermatids in both control and *Rfx2* KO mice. Broad domains are categorized into promoter (upper panel) and distal (bottom panel) groups based on their genomic locations;

e, Venn diagrams showing the number of overlapped broad H3K4me3 domains between control and *Rfx2* KO RS2 stage;

f, Snapshots of the UCSC genome browser showing the normalized ChIP-seq read densities of H3K4me3 in RS2 stage from control and *Rfx2* KO mice, over the *Cbx7* gene locus;

g, Co-immunoprecipitation showing that 2xFlag-RFX2 interact with HA-SETD1B in HEK293T cells;

h, Gross morphology of representative testes from an adult control and age-matched *Setd1b* *c*KO mutant (left panel). The testis/body weight ratio of control and *Setd1b* *c*KO mice was compared using the Student's *t*-test, ***$P < 0.001$ (3 mice per group). Data are presented as mean ± S.D. (right panel).

i, TEM analysis of sperm from adult control and *Setd1b* *c*KO cauda epididymis. Scale bar, 1 μm.

Supplementary Information Figure S13

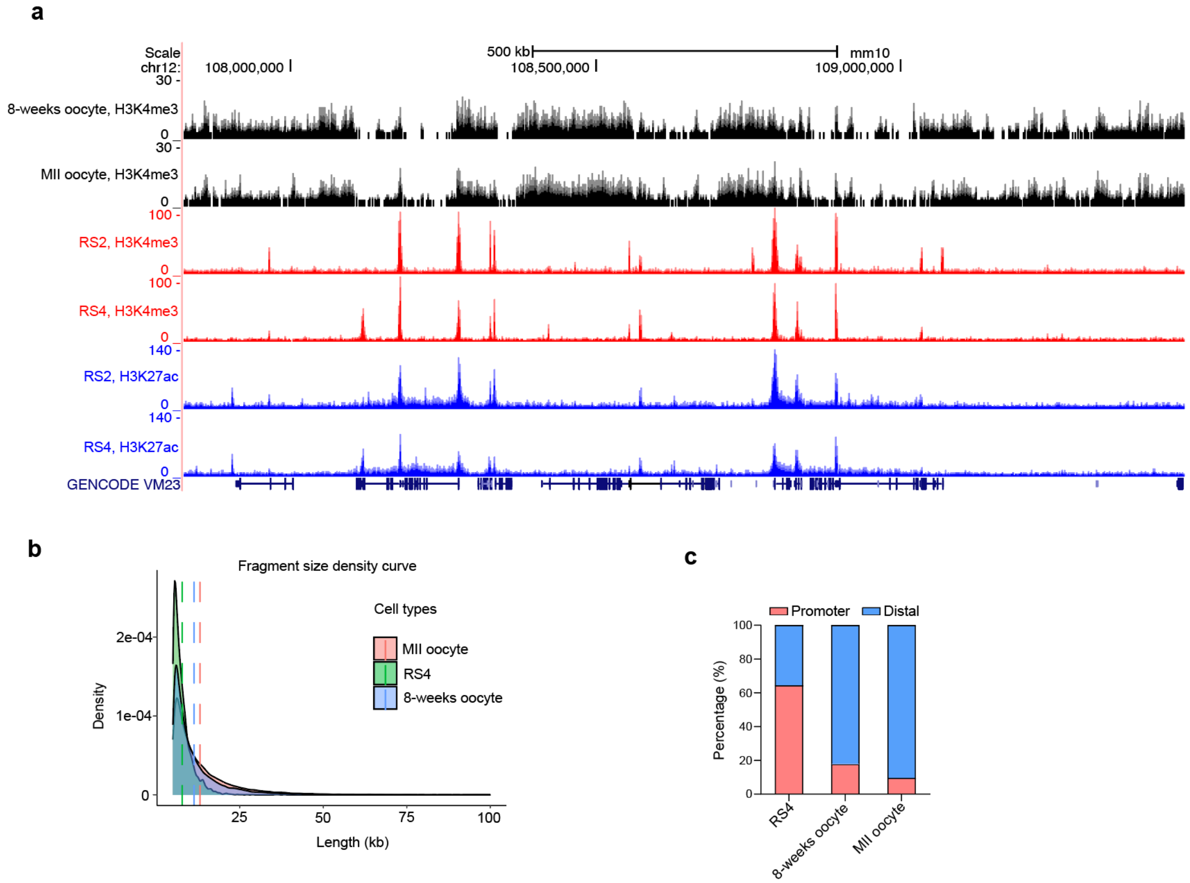

Supplementary Information, Fig. S13 Key Differences in Broad H3K4me3 Domains from RS and oocytes.

a, Snapshots of the UCSC genome browser showing the H3K4me3 and H3K27ac read density in oocyte and RS.

b, H3K4me3 peak width and density in oocyte and RS4 stage.

c, Genomic distribution of H3K4me3 peaks in RS4 and oocyte.
